# Emergence of genotype C1 Enterovirus A71 and its link with antigenic variation of virus in Taiwan

**DOI:** 10.1371/journal.ppat.1008857

**Published:** 2020-09-16

**Authors:** Kuan-Ying A. Huang, Peng-Nien Huang, Yhu-Chering Huang, Shu-Li Yang, Kuo-Chien Tsao, Cheng-Hsun Chiu, Shin-Ru Shih, Tzou-Yien Lin

**Affiliations:** 1 Division of Pediatric Infectious Diseases, Department of Pediatrics, Chang Gung Memorial Hospital, Taoyuan, Taiwan; 2 Research Center for Emerging Viral Infections, College of Medicine, Chang Gung University, Taoyuan, Taiwan; 3 Department of Medical Biotechnology and Laboratory Science, College of Medicine, Chang Gung University, Taoyuan, Taiwan; 4 Department of Laboratory Medicine, Chang Gung Memorial Hospital, Taoyuan, Taiwan; Purdue University, UNITED STATES

## Abstract

An outbreak of the hand-foot-mouth disease with severe neurological cases, mainly caused by the genotype C1 enterovirus A71 (EV-A71), occurred in Taiwan between 2018 and early 2019. In the recent decade, the most dominant EV-A71 genotypes in Taiwan were B5 and C4 but changed to C1 in 2018. Antibody-mediated immunity plays a key role in limiting the EV-A71 illness in humans. However, the level of neutralizing activities against genotype C1 virus by human polyclonal and monoclonal antibodies (MAbs) remains largely unclear. In the study, we demonstrated that that 39% (9 in 23) of post-infection sera from the genotype B5- or C4-infected patients in 2014–2017 exhibit reduced titers with the 2018–2019 genotype C1 viruses than with the earlier B5 and C4 viruses tested. This finding with polyclonal sera is confirmed with human MAbs derived from genotype B5 virus-infected individuals. The 2018–2019 genotype C1 virus is resistant to the majority of canyon-targeting human MAbs, which may be associated with the residue change near or at the bottom of the canyon region on the viral capsid. The remaining three antibodies (16-2-11B, 16-3-4D, and 17-1-12A), which target VP1 S241 on the 5-fold vertex, VP3 E81 on the 3-fold plateau and VP2 D84 on the 2-fold plateau of genotype C1 viral capsid, respectively, retained neutralizing activities with variable potencies. These neutralizing antibodies were also found to be protective against a lethal challenge of the 2018–2019 genotype C1 virus in an hSCARB2-transgenic mice model. These results indicate that the EV-A71-specific antibody response may consist of a fraction of poorly neutralizing antibodies against 2018–2019 genotype C1 viruses among a subset of previously infected individuals. Epitope mapping of protective antibodies that recognize the emerging genotype C1 virus has implications for anti-EV-A71 MAbs and the vaccine field.

## Author summary

EV-A71 is a cause of hand-foot-mouth disease, epidemics of which still regularly occur around the globe. Given that EV-A71 immune protection from the disease correlates with neutralizing antibody responses, but the responses in humans prior to an outbreak are still poorly understood. An outbreak of hand-foot-mouth disease among children emerged in Taiwan from 2018 to 2019, and genotype C1 EV-A71 caused most of the cases. Here, we characterized EV-A71-neutralizing antibody profiles in details at both the serological and monoclonal levels and showed that antibodies generated by humans prior to the emergence of genotype C1 EV-A71 less effectively neutralize C1 compared to the prior circulating genotypes, which implies the presence of antigenic variation in the EV-A71 genotypes. We further identified and mapped critical neutralizing epitopes of 2018–2019 genotype C1 EV-A71 on the top and margin of the viral capsid pentamer and demonstrated the *in vivo* protective effect of human monoclonal antibodies, which highlight the properties of human antibody-neutralizing sites on EV-A71 and the potential of human antibodies as antiviral agents.

## Introduction

Enterovirus A71 (EV-A71) is a major cause of hand-foot-mouth disease in children and is associated with severe neurological complications, including brain stem encephalitis and myelitis [1]. This virus is divided into seven distinct genogroups (A-G), of which two major genogroups, B and C, are further divided into B1-B5 and C1-C6 genotypes, respectively [2, 3]. Predominance of specific genotype is found in most outbreaks, but co-circulation of multiple genotypes can occur [4]. Genotypes B4, B5, and C4 viruses are mainly detected in the Asia-Pacific region, whereas genotypes C1 and C2 are prevalent in Europe [5, 6]. The genotype C1 EV-A71 emerged in Germany in 2015 and caused local outbreaks with severe neurological diseases in France, Poland, and Spain in recent years [7–14]. In Taiwan, previously circulating EV-A71 belonged to genotype C2 in 1998, B4 in 2000–2001, C4 in 2004–2005, B5 in 2008 and 2012, and C1 detected only rarely in 2009 [6]. An island-wide outbreak of hand-foot-mouth disease caused by EV-A71 affected Taiwanese children from 2018 to early 2019 and was associated with severe neurological disease (S1 Fig).

Preexisting neutralizing antibodies are critical for protection against severe complications and mortality caused by acute EV-A71 infection in humans [15–17]. Epidemiologic data has shown that the age-specific seropositive rate quickly rises from infancy (8%, 0.5–0.9 years) to preschool children (42%, 3–5.9 years) and reaches a plateau of approximately 60% among older children aged 6–11 years and above [15]. This indicates that young children are at greatest risk for novel EV-A71 infections. Preexisting antibody-mediated immunity to a certain extent may play a significant role in determining the prevalence of the virus. The importance of preexisting antibodies is further supported by protection from the acquisition of maternal antibodies against severe outcomes of acute EV-A71 infection in early infancy. The rate of symptomatic EV-A71 infection is lowest in seropositive infants who are younger than 6 months, and mortality and case fatality rates in this age group are also lower than those observed for children who were 0.5 to 1 year of age during the epidemic [15].

The capsid protein of EV-A71 is the major target when producing antibodies against viruses for immune system recognition and plays a key role in antigenicity [18–20]. An antigenic map was constructed based on serological data and showed antigenic diversity of different genotypes of EV-A71. The antigenic map showed that genotype B1 and B4 viruses are clustered closely together, genotype C2 and C4 form a separate cluster distinct from genotype B, and genotype B5 forms its own cluster [21]. This suggests a difference in antigenic properties and antigenic diversity among various genotypes of EV-A71 [21]. The epidemiological survey also supports that the emergence of new EV-A71 variants exhibiting altered antigenicity may result in increased circulation and altered clinical manifestations [14].

Here, we detected genotype C1 EV-A71 in children with hand-foot-mouth disease and severe neurological cases from 2018 to early 2019 during an EV-A71 outbreak in Taiwan. The virus is clustered to other genotype C1 viruses circulated in other endemic regions in the late 2010s. With the 2018–2019 genotype C1 viruses, we noted that a portion of post-infection sera collected from previously infected individuals in 2014–2017 had substantially reduced neutralizing titers. The majority of neutralizing human monoclonal antibodies (MAbs) that fail to react with the 2018–2019 genotype C1 viruses targets the epitope at the bottom of the capsid canyon region, where residue changes occurred in the 2018–2019 virus. A few antibodies that target the plateau and the 5-fold vertex epitopes retained neutralizing activities with the 2018–2019 virus and were protective *in vivo*. The neutralization epitopes of the genotype C1 EV-A71 capsid were identified with human antibodies, indicating that these epitopes are possible targets of antibody response upon natural infection and vaccination.

## Results

### Emergence of genotype C1 EV-A71 and its associations with neurological disease in 2018–2019

Twenty-one children with hand-foot-mouth disease were randomly enrolled from 2018 to early 2019. Acute EV-A71 infection was confirmed by positive viral isolations, positive reverse transcription polymerase chain reaction, and/or positive EV-A71-specific IgM rapid test results. Among the EV-A71 clinical isolates, 18 (90%) belonged to genotype C1 while the remaining 2 isolates belonged to genotype B5 by the VP1 sequence analysis (S1 Table).

The average age of enrolled patients was 4.3±2.9 (0.2–11.2) years and 67% of them were male. Eleven of the genotype C1 EV-A71-infected patients (11/18, 61%) experienced myoclonic jerk during acute illness, eight of whom had tachycardia and/or hypertension at rest and received intravenous immunoglobulin (S1 Table). Both genotype B5 EV-A71-infected patients had mild hand-foot-mouth disease without neurological manifestations in the study. There were no fatalities and all enrolled patients completely recovered during the study.

**Fig 1:**
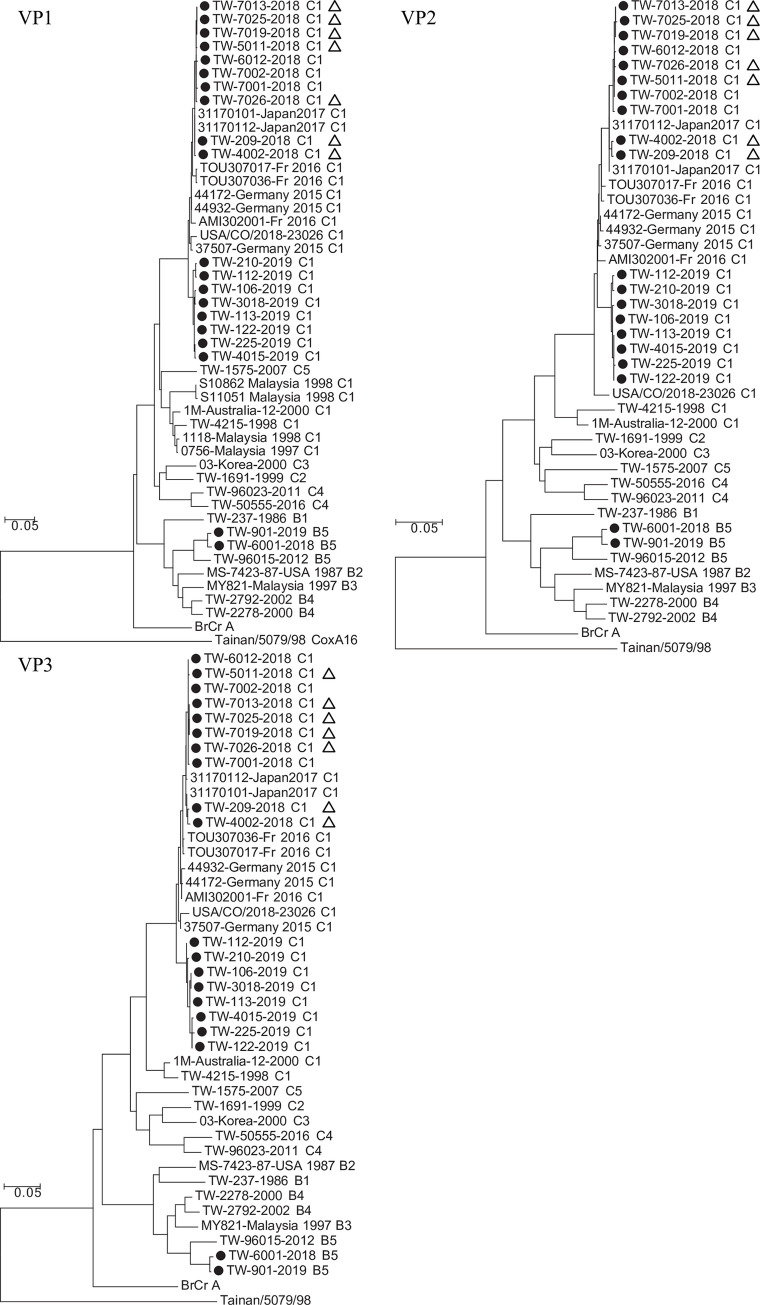
Phylogenetic tree of the EV-A71 isolates identified between 2018 and 2019 in Taiwan. Phylogenetic analyses were based on viral protein VP1, VP2 and VP3 nucleotide sequences of the Taiwan EV-A71 isolates identified between 2018 and 2019 (black circles) and a representative set of EV-A71 and enterovirus isolates (891 bases for VP1, 762 bases for VP2, 726 bases for VP3). The 2018–2019 EV-A71 that were isolated from patients with severe neurological manifestations were labeled with open triangles (refer to S1 Table). Trees were constructed by using the neighbor-joining method with 1,000 replicates through MEGA 7.0.25 (http://www.megasoftware.net/). Coxsackievirus A16 strain Tainan/5079/98 (AF177911.1) was used as the outgroup. The distances were computed using the Maximum Composite Likelihood method and are in the units of the number of base substitutions per site. Genotype assignment, country, and year of isolation are provided in the virus names. TW, Taiwan.

The VP1, VP2 and VP3 gene sequences of EV-A71 isolates were determined, and each isolate was assigned to a genotype by the phylogenetic analysis. In general, nucleotide sequence divergence in pairwise comparisons among Taiwan 2018–2019 EV-A71 isolates ranged from 0% to 18.6% (0.0%–4.0% amino acid divergence) for VP1, 0% to 18.4% (0.0%–2.8% amino acid divergence) for VP2, and 0% to 20.5% (0.0%–3.3% amino acid divergence) for VP3 (Fig 1). Eighteen sequences clustered with sequences of genotype C1 viruses isolated in Germany [8, 14], France [12], the USA [22] and Japan [6] (Fig 1, S2 Table). Our genotype C1 EV-A71 isolates formed two sub-clusters; the 2018 isolates were phylogenetically closer to the majority of isolates in Germany, France and Japan and the 2019 isolates formed another sub-cluster (Fig 1). The C1 genotype was associated with the outbreaks in Germany, France and the USA in terms of both severe and mild, sporadic cases in these regions. There was no association between distinct clusters with varying degrees of disease severity or manifestations in the analysis. Sequence analyses of Taiwan 2018–2019 genotype C1 EV-A71 revealed high nucleotide identities (97%–100% for VP1, 96%–99% for VP2, and 96%–99% for VP3) with recently circulating C1 viruses from GenBank, indicating the widespread circulation of this emergent EV-A71 variant [6, 8, 12, 14, 22].

### Reduced neutralizing antibody titers against 2018–2019 genotype C1 EV-A71 in convalescent sera

Antibody responses elicited by previous infection constitute one of the major components of humoral immunity. We measured the neutralizing activities against 2018–2019 genotype C1 EV-A71 in convalescent sera (median age 3.4 years; interquartile range 2–4 years) collected from 2014 to 2017 (S3 Table).

**Fig 2:**
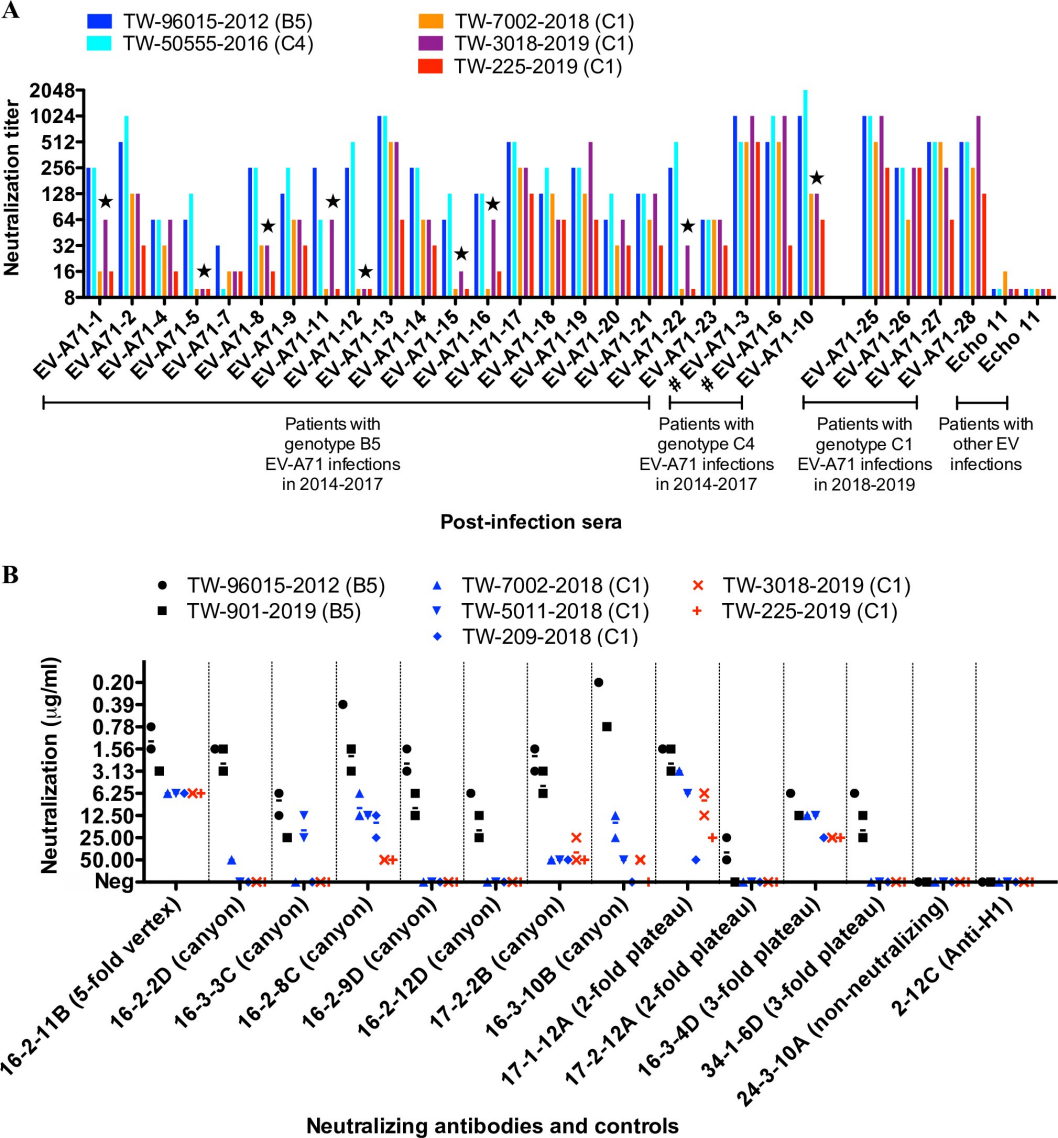
Neutralizing activities of human antibodies with the 2018–2019 genotype C1 EV-A71. (**A**) Neutralizing titers of polyclonal convalescent sera to EV-A71. A total of 23 sera were collected from hospitalized children with genotype B5 and C4 EV-A71 infection, 2014–2017 (refer to S3 Table). Sera from genotype C1 EV-A71- and echovirus 11-infected children were included as controls. Post-genotype C1 EV-A71 infection sera EV-A71-25, -26, -27, and -28 were obtained from subject 19, 20, 21, and 4, respectively (refer to S1 Table). Two post-genotype C4 EV-A71 infection sera (EV-A71-3 and -6) were obtained after intravenous immunoglobulin administration and marked with a hash sign. Each serum was assayed in triplicate for each virus with equivalent results. The serum that had an 8-fold decline in titers against the 2018–2019 genotype C1 EV-A71 compared to titers against the B5 and C4 virus is marked with a star. (**B**) Neutralizing activities of human monoclonal antibodies to EV-A71. Twelve neutralizing monoclonal antibodies that recognize the 5-fold vertex, canyon, 2-fold plateau, and 3-fold plateau epitopes on the viral capsid were tested. An EV-A71 capsid-targeting non-neutralizing antibody 24-3-10A and an influenza H1-targeting antibody 2-12C were included as controls. Each symbol represents an independent measurement. Some measurements overlap. The geometric mean concentration of neutralization is shown as a middle bar. In the neutralization assay, the failure of antibody up to 100 μg/ml to prevent the cytopathic effect was determined as no virus neutralizing activity. Each antibody was assayed in duplicate for each virus with equivalent results and the assay was carried out three times. 16-2-11B, 17-1-12A, and 16-3-4D retained neutralizing activities against the majority of 2018–2019 genotype C1 EV-A71 and the analysis showed < 4-fold change in neutralization potencies for genotypes B5 and C1.

At first, sera from hospitalized children in the 2018–2019 outbreak were included as controls. All of them had strong neutralizing titers (1:64 to 1:1024) against 2018–2019 genotype C1 viruses, indicating the development of genotype-specific neutralizing antibodies upon natural infection (Fig 2A). In contrast, two sera from echovirus 11-infected children had low titers against all EV-A71 viruses tested.

In all, 23 sera collected from 2014 to 2017 were tested, of which 20 and 3 sera were from hospitalized children with genotypes B5 and C4 EV-A71 infection, respectively (S3 Table). Since 2004, genotypes B5 and C4 EV-A71 have been the predominant genotypes circulating in Taiwan, causing major outbreaks in 2010–2011 and 2011–2012, and co-circulation of the two genotypes resulted in small-scale outbreaks in the following years [6, 23]. Fig 2A shows that eight sera from the B5 group (8/20, 40%) and one serum from the C4 group (1/3, 33%) exhibited ≥ 8-fold lower titers against the 2018–2019 genotype C1 EV-A71 compared to titers against the B5 and C4 viruses tested. Of the low-titer sera for 2018–2019 EV-A71, three (EV-A71-5, EV-A71-12, and EV-A71-15) showed titers below the protective level (1:8 to 1:16) [17], while all three had strong titers for B5 (1:64 to 1:256) and C4 (1:128 to 1:512) viruses. These substantial changes in the neutralizing titers of post-infection sera suggest alterations in the antigenicity of 2018–2019 EV-A71 variants.

### Loss of activity of canyon-targeting antibodies against 2018–2019 genotype C1 EV-A71

We found reduced neutralizing titers with genotype C1 EV-A71 in a subset of convalescent sera post genotype B5 or C4 virus infection. To examine the activity of neutralizing antibodies at the clonal level, a panel of human MAbs was tested against the 2018–2019 genotype C1 EV-A71. Twelve MAbs were previously isolated from genotype B5 EV-A71-infected patients and represented neutralizing antibodies that target the 5-fold vertex, canyon, 3-fold plateau, and 2-fold plateau epitopes on the viral capsid [24]. The majority of MAbs (5 of 7 canyon MAbs, 1 of 2 3-fold plateau MAbs, 1 of 2 2-fold plateau MAbs) displayed little or no activities against the 2018–2019 genotype C1 EV-A71 (Fig 2B). 16-2-8C and 16-3-10B were two most potent anti-canyon antibodies against the genotype B5 EV-A71, but both exhibited greatly reduced activities against the 2018–2019 genotype C1 EV-A71 (neutralizing concentrations against B5 v.s. C1 for 16-2-8C, 1.24±1.09 μg/ml v.s. 27.92±19.03 μg/ml, p = 0.0005; for 16-3-10B, 0.49±0.32 μg/ml v.s. 38.89±17.05 μg/ml, p = 0.0017; two-tailed Mann–Whitney U test).

The remaining MAbs, 16-2-11B, 17-1-12A and 16-3-4D, retained variable potencies. 16-2-11B was the most potent antibody against genotype C1 EV-A71 and had similar activities against genotypes B5, C4, and C1.

**Fig 3:**
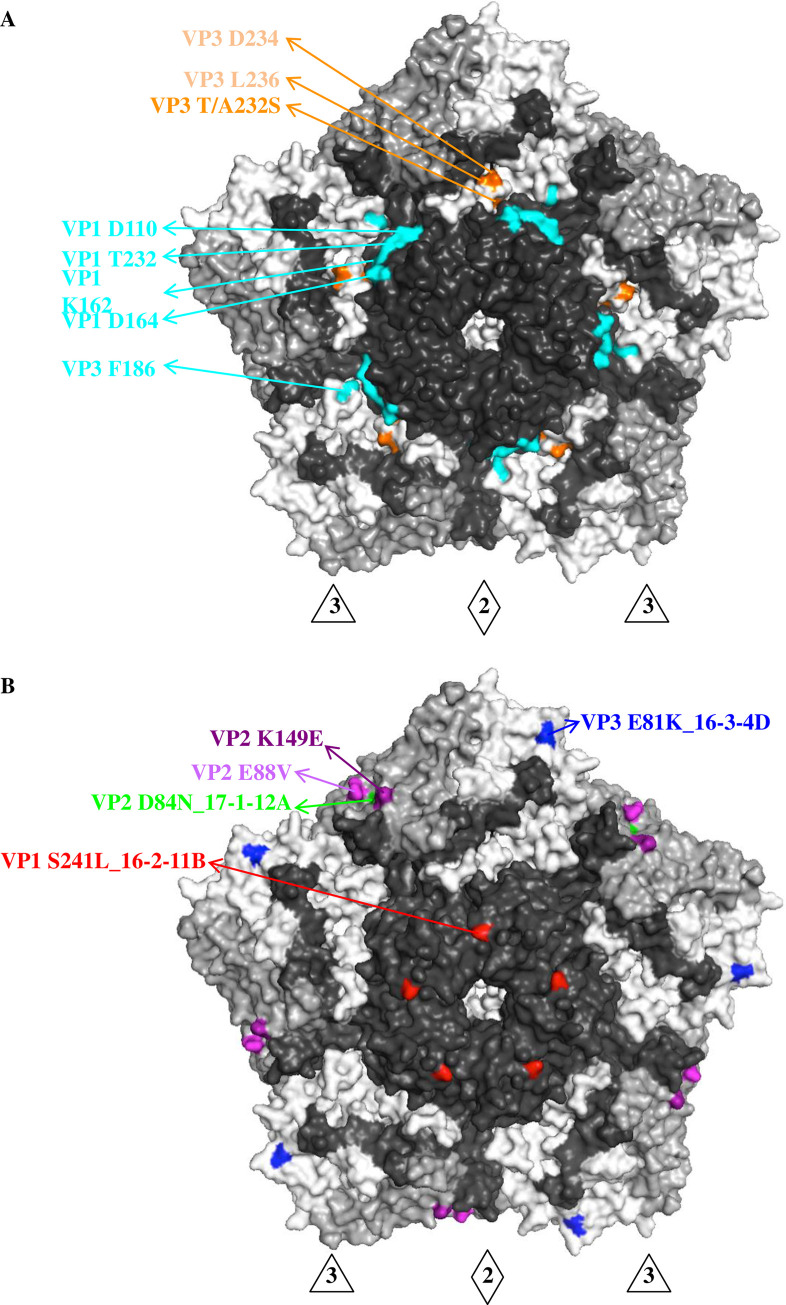
Antigenic determinants on the viral capsid of genotype C1 EV-A71. (**A**) Canyon epitopes, including VP1 D110, VP1 T232, VP1 K162, VP1 D164 and VP3 F186, recognized by human anti-EV-A71 MAbs 16-2-9D, 16-2-12D, 16-3-3C and 16-2-2D, were colored in cyan [24]. The surface residue 232 of VP3, located at the canyon region of capsid, was distinct in genotype C1 EV-A71 (S2 Fig) and interacted with VP3 D234 and L236 canyon residues (colored in orange). (**B**) Epitope mapping on the viral capsid by genotype C1 EV-A71-neutralizing MAbs. Escape variants were selected using MAbs 16-2-11B, 16-3-4D and 17-1-12A and revealed single amino acid substitutions at capsid residues VP1 S241 (colored in red), VP3 E81 (colored in blue) and VP2 D84 (colored in green), respectively. VP2 E88V and VP2 K149E were previously identified in the escape variant of genotype B5 and C4 EV-A71 with MAb 17-1-12A [24]. The surface view of pentamer shown with the 5-fold vertex at the center are created using the software program PyMOL (PDB 3VBS). The capsid VP1 protein is colored in black, VP2 colored in grey and VP3 colored in white. Abbreviations: 3, 3-fold axis; 2, 2-fold axis.

Sequence alignment of the 2018–2019 genotype C1 EV-A71 with previous B5 and C4 EV-A71 showed five residue changes on the viral capsid (S2 Fig), of which VP3 T/A232S was on the surface and interacted with adjacent surface residues VP3 D234 and VP3 L236. These residues were located at the bottom of the capsid canyon and adjacent to the epitope recognized by 16-2-9D, 16-2-12D, 16-3-3C, and 16-2-2D [24] (Fig 3A). Structural mapping indicated that residue changes on the capsid of the 2018–2019 genotype C1 EV-A71 may affect specific recognition by canyon-targeting antibodies.

### Critical determinants of the 2018–2019 genotype C1 EV-A71 capsid

To explore the antigenic determinants of the genotype C1 EV-A71 capsid engaged by human neutralizing antibodies, we selected escape variants from TW-225-2019 in the presence of MAb 16-2-11B, 17-1-12A, or 16-3-4D *in vitro*. Substitutions in escape variants were compared to the parental viruses and are shown in Fig 3B. Each MAb-escape variant has a single residue mutation on the viral capsid that greatly reduces the neutralizing and binding activities of the antibody (Fig 3B, S3 Fig).

Two capsid residues of escape variants selected by MAbs 16-2-11B and 16-3-4D were identical to previously identified epitopes on the C4 and B5 viruses [24]. VP1 S241 is targeted by MAb 16-2-11B and located on the 5-fold vertex of genotype C1 virus, which has been proposed as an attachment site for cellular heparan sulfate [25]. VP3 E81 is targeted by MAb 16-3-4D near the 3-fold axis of viral capsid and in the BC loop of VP3, one of the dominant neutralizing epitopes for picornaviruses [26, 27].

MAb 17-1-12A selected a single mutation, VP2 D84N, which is near the 2-fold axis of the viral capsid. Previously, 17-1-12A selected single substitutions VP2 E88V and VP2 K149E in the C4 and B5 escape variants, respectively [24]. We mapped these three VP2 residues that are critical for viral neutralization onto the capsid and found that they lie close to each other in the structure (Fig 3B).

### *In vivo* protection of neutralizing antibodies against 2018–2019 genotype C1 EV-A71

To examine the *in vivo* protection of genotype C1 EV-A71-neutralizing MAbs 16-2-11B, 17-1-12A, and 16-3-4D, we established a mouse infection model, in which three-week-old human scavenger receptor class B member 2 (hSCARB2)-transgenic mice (C57BL/6 background) were infected intraperitoneally with 2018–2019 EV-A71 (TW-5011-2018) [28].

**Fig 4:**
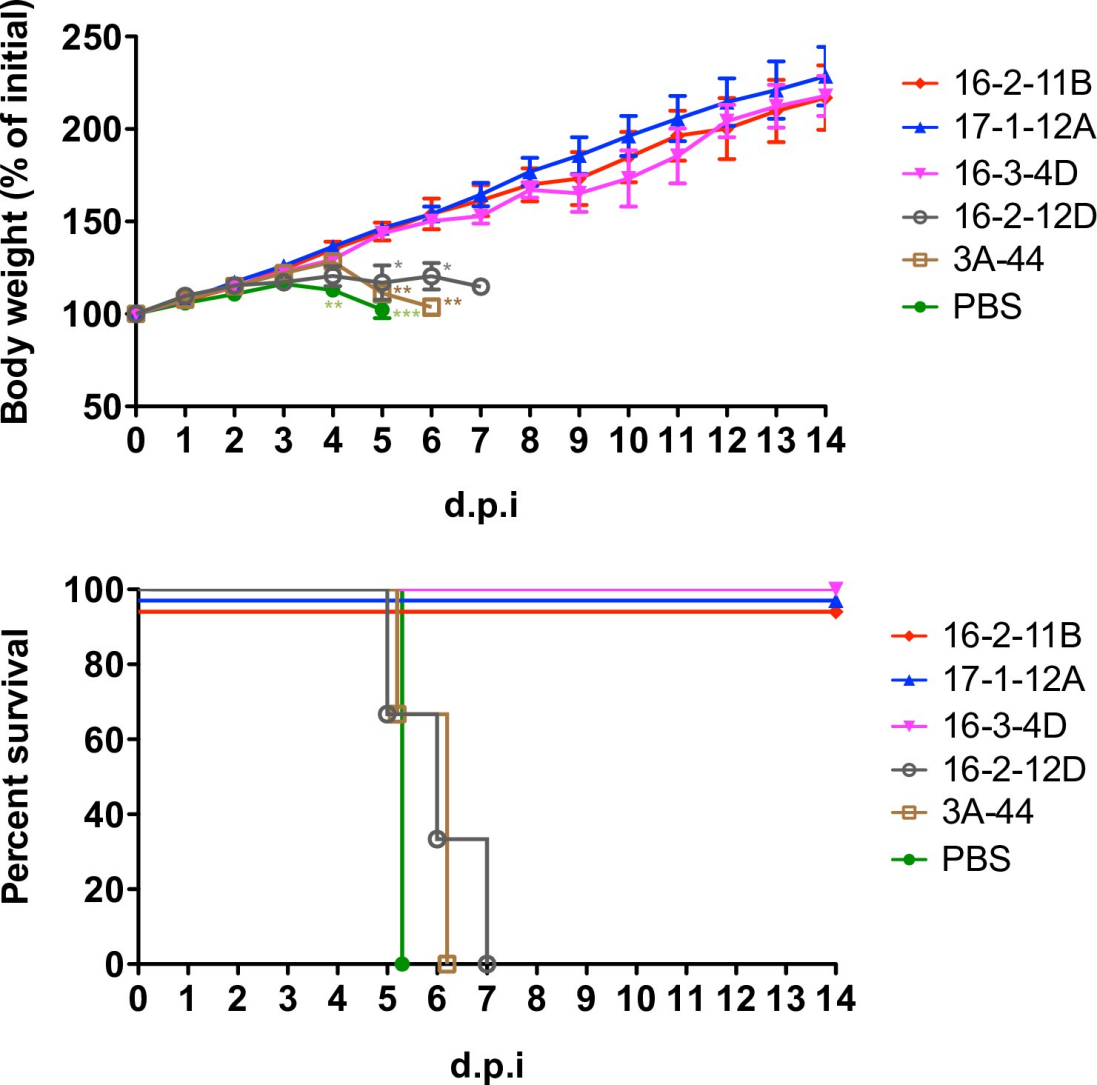
The *in vivo* protection by genotype C1 EV-A71-neutralizing MAbs. The hSCARB2-transgenic mice were administered with 16-2-11B, 17-1-12A, and 16-3-4D at 10 mg per Kg 24 hours prior to a lethal challenge of genotype C1 EV-A71 TW-5011-2018 (n = 3 per group). Weight change following infection and survival rate were measured. Non-neutralizing anti-EV-A71 canyon MAb 16-2-12D and anti-avian influenza H7 MAb 3A-44 were human IgG controls. The weight data are presented as the means ± standard error of the mean and the comparison of weight change among groups was analyzed using one-way ANOVA followed by Tukey’s post hoc analysis. *, p < 0.05; **, p < 0.01; ***, p < 0.001. d.p.i, day post infection.

Fig 4 shows that MAbs 16-2-11B, 17-1-12A, and 16-3-4D protected 100% of mice from a viral challenge with 10 times the 50% lethal dose (LD50) of virus when administered at a dose of 10 mg per kg 24 hours before infection. They also prevented any weight loss, whereas mice that received phosphate-buffered saline (PBS) and IgG controls displayed significant weight loss by day 5 (day 5 body weight, p < 0.001 for the comparison of PBS and neutralizing MAb groups; p < 0.01 for the comparison of 3A-44 and neutralizing MAb groups; p < 0.05 for the comparison of 16-2-12D and neutralizing MAb groups; one-way ANOVA followed by Tukey’s post hoc analysis). The human MAb 3A-44 to influenza H7 and human MAb 16-2-12D to EV-A71 canyon provided IgG controls in the experiment and were not protective for the infected mice.

## Discussion

The genotype C1 EV-A71 emerged and became the leading cause of hand-foot-mouth disease and severe neurological complications in Taiwan from 2018 to early 2019. The emergence or re-emergence of an EV-A71 outbreak among young children could be a consequence of the accumulation of susceptible individuals who are not protected by immune memory or are still naïve to the virus. The seropositivity rate varied greatly among different endemic regions and reflected the disease burden in the local area [29, 30]. Young children who are seronegative to EV-A71 have a high risk of developing acute illness and spreading the virus during an outbreak. On the other hand, those who were previously infected would have acquired a certain level of humoral memory but may have impaired protection against newly emerging variants. The genetic evolution of EV-A71 is likely driven by selective pressure exerted by humoral immune response. Recent studies have also reported that inter-genotype shifts are common in Taiwan and Japan between EV-A71 outbreaks [31]. Cross-neutralization by anti-EV-A71 antibodies was observed among genotypes using animal and human antisera [32, 33]. However, children infected with genotype B had higher neutralization titers against genogroup B than genogroup C [34]. A previous vaccine trial also showed that the genotype B4 EV-A71 vaccine fails to elicit a protective neutralization titer against genotype C2 [35]. Such evidence clearly suggests the existence of antigenic variation among EV-A71 genotypes, although the underlying mechanism determining viral emergence and the scale and severity of hand-foot-mouth disease outbreak may be a complex interplay between cross-immunity, pathogen evolution, and herd immunity.

An alternative possibility for the EV-A71 outbreak is that the recombination event may contribute to the acquisition of distinct antigenicity or pathogenicity for a newly emerging variant. A recent example is the emerging genotype C1 EV-A71 associated neurologic complications in several outbreaks in Europe [3, 8, 12]. This new EV-A71 strain has a genotype C1-like VP1 region but has genetic recombination patterns with B3/C2-like and C4 viruses in the 5´ untranslated and the P2/P3 regions, respectively. It is suggested that this new EV-A71 strain may have been generated by recombination of the locally circulating C1 strain with the imported C4 strain that recently became dominant [23, 36].

Human sera and MAb derived from infected individuals were used to characterize the antigenic phenotype of the 2018–2019 genotype C1 EV-A71. We found that canyon-targeting antibodies, especially those that bind to the bottom of the canyon, lost neutralizing activity against the 2018–2019 genotype C1 EV-A71. The canyon region is believed to be poorly immunogenic to evoke a picornavirus-reactive antibody response because of its relatively inaccessible structure on the viral surface. The exemption from antibody-mediated attack would permit the conservation of canyon residues, which ideally offers the virus a functional fitness for binding to the host cell receptor [37, 38]. Sequence alignment showed that the canyon epitope is highly conserved among circulating EV-A71 strains except for one surface residue adjacent to the epitope, VP3 residue 232, mutates (threonine or alanine-to-serine substitutions) in 2018–2019 genotype C1 viruses. Previously, we have showed that single amino acid substitution in the epitope would allow for the escape of EV-A71 from neutralization by human antibodies [24]. Moreover, the differences in length and properties of the side chain may influence the steric hindrance between neighboring residues and lead to the changes in overall tertiary structure of the epitope region.

Critical neutralization determinants of genotype C1 EV-A71 were mapped on the 5-fold vertex and plateau region of the viral capsid. All three residues (VP1 Ser 241, VP3 Glu 81, VP2 Asp 84) are conserved in circulating EV-A71 strains. The VP1 residue 241 is adjacent to the receptor binding site and is proposed to engage with cellular receptor P-selectin glycoprotein ligand-1 and heparan sulfate [25]. Chang et al demonstrated that amino acid substitutions of VP1 residues 145, 146, and 241 on the 5-fold vertex affect the binding of EV-A71 to the cellular receptor and subsequently viral entry into the cell [39], indicating the role of the 5-fold vertex in the initiation of viral infection. VP3 residue 81 and VP2 residue 84 are part of the 3-fold and 2-fold plateau epitopes recognized by human neutralizing antibodies, respectively. VP3 residue 81 was also found within the footprints of two murine-derived neutralizing antibodies, D6 and A9 [19], both of which are potent neutralizer that prevent the virus from attaching. Thus, VP3 residue 81 appears to be immunogenic to elicit a neutralizing antibody response in both humans and animals. VP2 residue 84 is near the VP2 GH loop in the 3D structure, where several residues (e.g., VP2 Lys 149) are involved in viral attachment with cellular hSCARB2 [40]. Taken together, antibody epitopes on the genotype C1 EV-A71 capsid may overlap or are in close proximity to key residues that mediate the attachment of the virus to host cell receptors.

Following the eradication of polio in developed countries, EV-A71 has become one of the major enteroviruses that tend to cause severe neurological complications and mortality. Currently, there is no available antiviral agent and no licensed vaccine in most areas with endemic EV-A71. Supportive care is the mainstay of clinical management for symptomatic infections. There is an urgent need for the development and clinical application of novel agents for the prevention and treatment of acute EV-A71 infection. Lim et al showed that passive transfer of neutralizing MAbs against EV-A71 surface proteins prevents the development of severe illness in challenged mice [41]. Adoptive transfer of neutralizing antibodies has also been found to contribute to reductions in mortality rate and tissue viral load in B-cell deficiency mice [42]. In humans, clinical observations of passive therapy in immunocompromised patients support the role of neutralizing antibodies in treating severe enteroviral infections [43, 44]. Here, we provided *in vivo* evidence that human neutralizing MAbs to the EV-A71 capsid protect against a lethal virus challenge in a susceptible mouse model, indicating the potential prophylactic value of potent and broadly reactive human MAbs in clinical settings.

## Methods

### Ethical approval

The study protocol and informed consent were approved by the ethics committee at the Chang Gung Memorial Hospital. Each subject provided written informed consent. The study and all associated methods were carried out in accordance with the approved protocol, the Declaration of Helsinki and Good Clinical Practice guidelines.

### Samples

Hospitalized children with hand-foot-mouth disease or herpangina were randomly enrolled at the Chang Gung Memorial Hospital in 2018–2019. The laboratory diagnosis for acute EV-A71 infection was based on positive reverse-transcriptase polymerase chain reaction [45] and/or positive EV-A71-specific IgM rapid test (Formosa One Sure EV71 IgM Rapid Test kit, Formosa Biomedical Technology, Taiwan) [45] and/or isolation of virus from throat or rectal swabs. Sera were collected and stored in -80°C before test.

### Viruses

EV-A71 clinical strains, including TW-96015-2012, TW-50555-2016, TW-209-2018, TW-5011-2018, TW-7002-2018, TW-901-2019, TW-3018-2019 and TW-225-2019, were used in the experiments. All viruses were plaque purified and amplified in rhabdomyosarcoma cells.

All of the throat and/or rectal swab specimens were submitted to isolate enterovirus and rhabdomyosarcoma cells were used for virus isolation and propagation. Those positive for enteroviruses were examined by type-specific monoclonal antibodies against EV-A71 for serotype identification in the immunofluorescent assay [46]. Viral RNA was extracted using a QIAamp Viral RNA Extraction Kit (Qiagen, Germany) and the VP1, VP2 and VP3 regions were amplified with a set of primers (S4 Table). The analysis of VP1,VP2 and VP3 sequences was carried out by comparisons with reference sequences in GenBank using the Basic Local Alignment Search Tool available at the U.S. National Center of Biotechnology Information. The sequences were then aligned using ClustalW included in MEGA7 [47].

### Monoclonal antibodies

Antibodies were isolated from human individuals who were naturally infected with EV-A71 in Taiwan as previously described [24]. Heavy and light chain plasmids were transfected into 293T cells for human monoclonal antibody expression. Representative antibodies were expanded and purified using protein A-sepharose (Merck, Germany).

### Neutralization of serum and monoclonal antibody with enteroviruses

Neutralizing titers of sera and monoclonal antibodies with EV-A71 were measured by a method as previously described [24]. Briefly, serially diluted samples were incubated with an equal volume of 100 TCID_50_ virus at 37°C for 2 hours. Then, the rhabdomyosarcoma cell suspension was added to the virus-antibody mixture and incubated at 37°C for 4–5 days. For each assay, cell controls and virus back-titration were setup. Cytopathic effect was examined before and after staining of crystal violet. The neutralization titer is defined as the reciprocal of highest dilution for which the cytopathic effect was prevented in triplicate culture wells.

### Selection of escape mutants with human monoclonal antibodies

Wild-type plaque-purified EV-A71 were diluted to 50 TCID_50_ times neutralization titer against tested monoclonal antibody of 25 μg/ml and incubated with an equal volume of monoclonal antibody at a final concentration of 25 μg/ml for 1 hour at room temperature. The mixture was then added to the flat-bottomed well containing a confluent monolayer of rhabdomyosarcoma cells and incubated at 37°C for 5 days. If there was no cytopathic effect observed, the cells and supernatant were collected, freeze-thawed three times, filtered, and then re-infected a fresh preparation of RD cell layer at 37°C for 4 days. Once cytopathic effect of cells was observed at first or second re-infection cycle, the cells and supernatant were collected and freeze-thawed three times. Cell debris was removed by centrifugation, virus-containing solution was harvested and then the virus was plaque-purified. In the study, one to two re-infection cycles were needed for the presence of cytopathic effect and the development of mAb-resistant mutants.

Plaque-purified antibody-resistant mutants were confirmed by testing the abolishment of binding in the flow cytometry-based binding assay and neutralization in the cytopathic effect-based neutralization assay by antibody. Neutralizing activities of monoclonal antibodies with EV-A71 were measured by a method as previously described [24].

The flow cytometry-based binding assay was performed as previously described [24]. A confluent monolayer of RD cells were incubated with the optimized infectious dose of EV-A71 one day before the experiment. The next day, the cells were harvested, washed, and resuspended. Fixed and permeabilized cells were blocked with saponin-3% BSA. After blocking, cells were incubated with purified monoclonal antibodies (5 μg/ml) in BD Perm/Wash buffer and mouse IgG antibody to EV-A71 3C (1 μg/ml)(GeneTex, USA). Bound antibodies were detected with fluorescence-conjugated anti-IgG secondary antibodies (Goat anti-human and Goat anti-mouse IgG) (Thermo Fisher Scientific, USA) in BD Perm/Wash buffer. Cells were analyzed with a BD FACSCanto II flow cytometer. Results were derived from an analysis of 10,000 gated events of EV-A71-infected (3C-positive) cells and are shown as the percentage of EV-A71-infected cells that bound human anti-EV-A71 antibodies. Mock-infected RD cells were used as an antigen control, and the EV-A71 convalescent serum, an anti-EV-A71 VP2 mouse monoclonal antibody MAB979 (EMD Millipore, USA) and PBS were used as antibody controls for each experiment.

Viral RNAs of escape mutants and parental EV-A71 strain TW-225-2019 (genotype C1) were isolated using a QIAamp Viral RNA Mini Kit (QIAGEN, Germany) according to the manufacturer’s protocol. RNA was reverse-transcribed to cDNA with FL-R primer (5’- TTTTTTTTTTGCTATTCYGGTTATAACAAAT-3’) using a ReverTra Ace cDNA synthesis kit (TOYOBO, Japan) according to the manufacturer’s protocol. Polymerase chain reactions were carried out for amplification of the VP4, VP2, VP3 and VP1 genes using a KOD -Plus- kit (TOYOBO, Japan) according to the manufacturer’s protocol. The primers used for the cDNA amplification were as follows: C1-VP4-F: 5’-ATCCGGTGTCTAACAGAGC-3’, C1-VP4-R: 5’-CACTCACCATAGCCAACTAT-3’; C1-VP2-F: 5’-ACAGAGCCTTAAACAAGATCCAGAT-3’, C1-VP2-R: 5’-AAAGTTCGGCAGGATGGGTGC-3’; C1-VP3-F: 5’-TTCGACCAAGGAGCGACAC-3’, C1-VP3-R: 5’-CTGTAGGCGCTGGTAAAGC-3’; C1-VP1-F: 5’-GCAGCAGCCCAGAAAAA-3’, C1-VP1-R: 5’-AAGGTTTGCCCAATCATTGTG-3’. PCR products were gel purified. The Sanger method was used to for direct sequencing of the VP4, VP2, VP3 and VP1 DNA products. The primers for sequencing were the same primers used for polymerase chain reactions. The VP4, VP2, VP3 and VP1 sequences of escape mutants were compared with those of parental virus to identify point mutations.

### *In vivo* animal study

All animal studies were performed using protocols approved by the Chang Gung University Animal Care Institutional Review Board and in accordance with the ‘Guide for the care and use of laboratory animals’, the recommendations of the Institute for Laboratory Animal Research, and Association for Assessment and Accreditation of Laboratory Animal Care International Standards.

The hSCARB2-transgenic mice (C57BL/6 background) were maintained under specific pathogen-free conditions in the animal facilities of National Laboratory Animal Center and Chang Gung University, Taiwan. Three-week-old mice were randomly divided into groups and infected intraperitoneally with 10 times 50% lethal dose of EV-A71 24 hours after an intraperitoneal administration of monoclonal antibody at a dose of 10 mg/kg. Mice were weighed daily and mice with 20% weight loss were humanely killed.

### Statistics

The TCID_50_ in the rhabdomyosarcoma cell line for EV-A71 were calculated by Reed-Muench method. The neutralizing concentrations of MAbs with the genotype B5 and C1 EV-A71 was compared using the two-tailed Mann–Whitney U test. The binding activity of MAbs with EV-A71 in the flow cytometry-based binding assay was compared between groups using the two-tailed Mann–Whitney U test. In the mice challenge study, the weight change following infection was measured and the comparison of weight change among groups was analyzed using one-way ANOVA followed by Tukey’s post hoc analysis. A p value of less than 0.05 was considered significant. Graphs were presented by Microsoft Excel and GraphPad Prism software.

## Supporting information

S1 Fig An outbreak of EV-A71 in Taiwan, January 2018 to March 2019. A total of 40 enterovirus-associated severe cases and the percentage of EV-A71 in all enterovirus isolates were reported based on the retrieved data from Taiwan National Infectious Disease Statistics System of Taiwan Centers for Disease Control (https://nidss.cdc.gov.tw), Jan 2018-Mar 2019. Severe cases were defined as the presence of encephalitis and/or autonomic nervous system dysregulation and/or cardiopulmonary failure and/or mortality [1].
(TIF)

S2 Fig Amino acid sequence alignment of VP1, VP2 and VP3 of EV-A71. The sequences of genotype B5 (TW-96015-2012), C4 (TW-50555-2016) and C1 EV-A71 (TW-7002-2018, TW-3018-2019, TW-225-2019) were analyzed. The numbers correspond to the amino acid positions in the mature EV-A71 capsid. Five residue changes found in 2018–2019 genotype C1 EV-A71 were marked by rectangle and the residue located on the capsid surface was marked by red rectangle.
(TIF)

S3 Fig Binding and neutralizing activities of genotype C1 EV-A71-neutralizing monoclonal antibodies with escape variants. The binding and neutralizing activities were examined by flow cytometry-based binding and cytopathic effect-based neutralization assays, respectively. The binding data were derived from an analysis of 10,000 gated events of EV-A71-infected cells and shown as the percentage of EV-A71-infected cells that bound anti-EV-A71 monoclonal antibodies. The non-neutralizing anti-EV-A71 capsid MAb 16-2-1A was unaffected by any of escape variants [24]. Data are presented as the mean ± standard error of the mean and represent four independent experiments (n = 4). The binding activity was compared between two groups using the two-tailed Mann–Whitney U test. ns, not significant, *: p value <0.05. In the neutralization assay, the failure of antibody up to 100 μg/ml to prevent the cytopathic effect was determined as no virus neutralizing activity. ND, not determined.
(TIF)

S1 Table Demographic and clinical characteristics of enrolled patients with acute EV-A71 infections from 2018 to early 2019. (PDF)

S2 Table The complete VP2-VP3-VP1 sequences of enteroviruses. (DOCX)

S3 Table Convalescent sera from patients with acute EV-A71 infections, 2014–2017. (PDF)

S4 Table Primers for sequencing of VP1, VP2 and VP3 of EV-A71 in the study. (PDF)
