# Supplementary figures and images for: Emergence of genotype C1 Enterovirus A71 and its link with antigenic variation of virus in Taiwan
Source: PLoS Pathog. 2020 Sep 16;16(9):e1008857. doi: 10.1371/journal.ppat.1008857 (PMC7521691; doi:10.1371/journal.ppat.1008857)

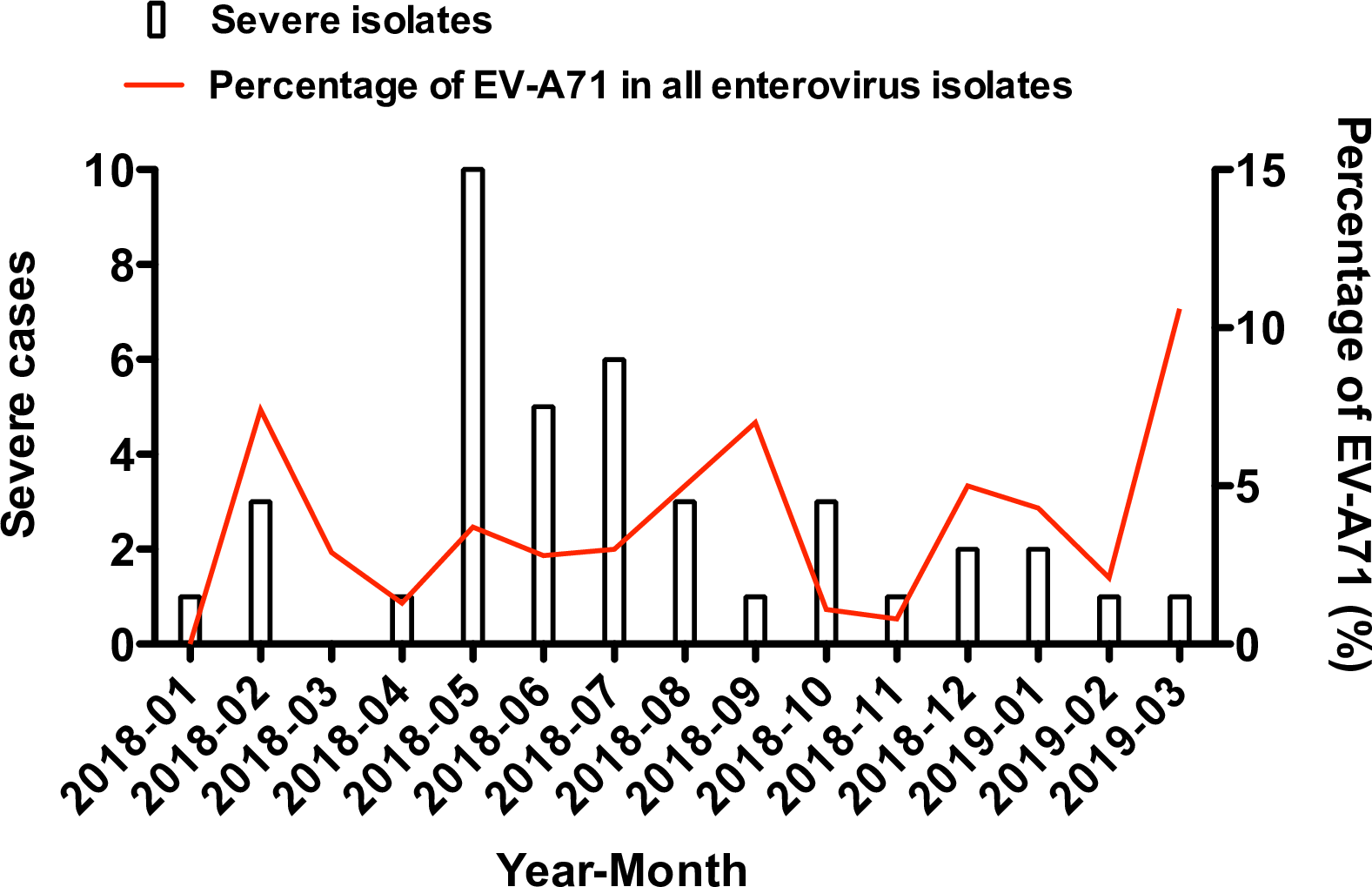

Supplement: S1 Fig — A total of 40 enterovirus-associated severe cases and the percentage of EV-A71 in all enterovirus isolates were reported based on the retrieved data from Taiwan National Infectious Disease Statistics System of Taiwan Centers for Disease Control (https://nidss.cdc.gov.tw), Jan 2018-Mar 2019. Severe cases were defined as the presence of encephalitis and/or autonomic nervous system dysregulation and/or cardiopulmonary failure and/or mortality [1]. (TIF) [file ppat.1008857.s001.tif]

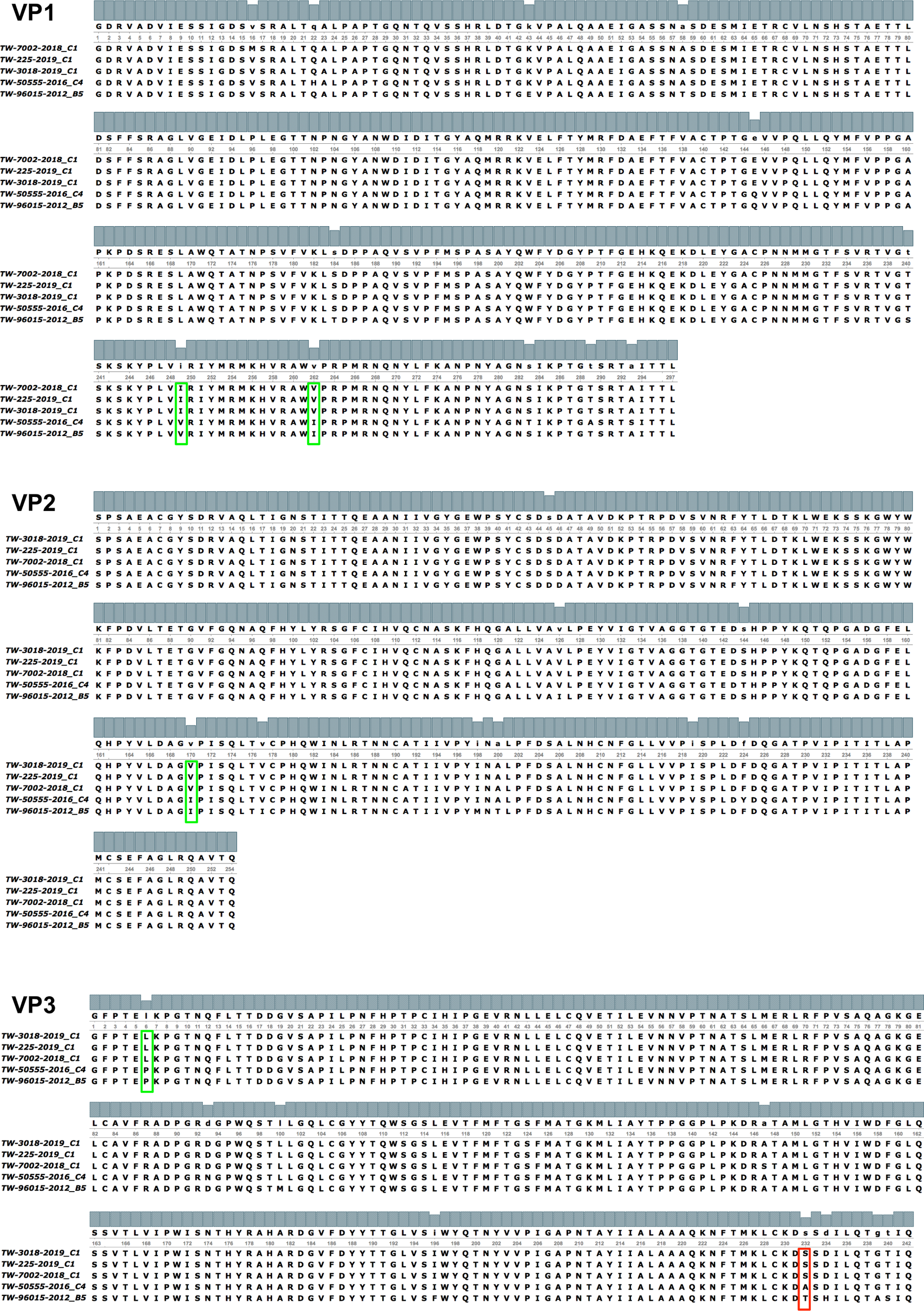

Supplement: S2 Fig — The sequences of genotype B5 (TW-96015-2012), C4 (TW-50555-2016) and C1 EV-A71 (TW-7002-2018, TW-3018-2019, TW-225-2019) were analyzed. The numbers correspond to the amino acid positions in the mature EV-A71 capsid. Five residue changes found in 2018–2019 genotype C1 EV-A71 were marked by rectangle and the residue located on the capsid surface was marked by red rectangle. (TIF) [file ppat.1008857.s002.tif]

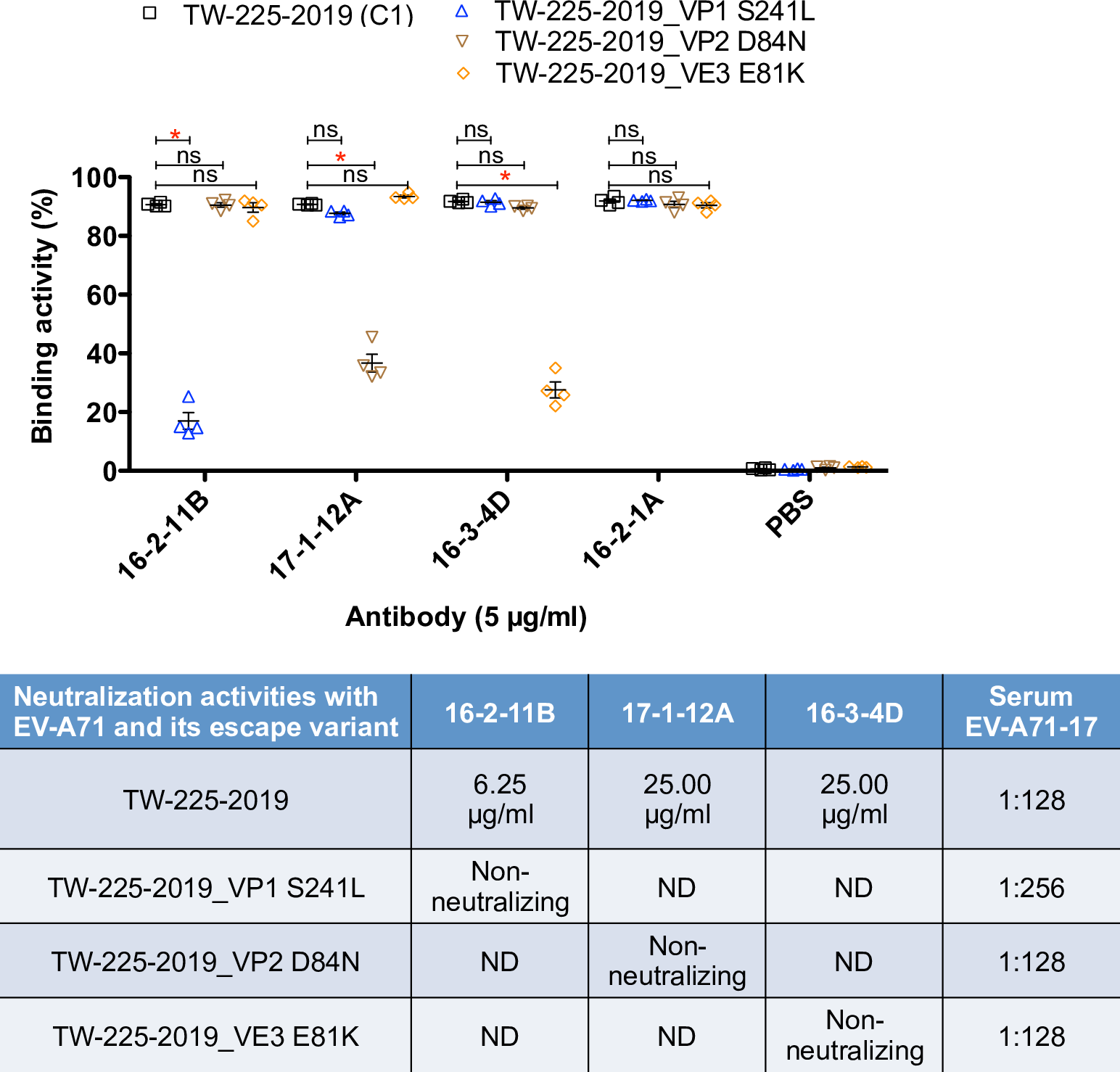

Supplement: S3 Fig — The binding and neutralizing activities were examined by flow cytometry-based binding and cytopathic effect-based neutralization assays, respectively. The binding data were derived from an analysis of 10,000 gated events of EV-A71-infected cells and shown as the percentage of EV-A71-infected cells that bound anti-EV-A71 monoclonal antibodies. The non-neutralizing anti-EV-A71 capsid MAb 16-2-1A was unaffected by any of escape variants [24]. Data are presented as the mean ± standard error of the mean and represent four independent experiments (n = 4). The binding activity was compared between two groups using the two-tailed Mann–Whitney U test. ns, not significant, *: p value <0.05. In the neutralization assay, the failure of antibody up to 100 μg/ml to prevent the cytopathic effect was determined as no virus neutralizing activity. ND, not determined. (TIF) [file ppat.1008857.s003.tif]
