# Supplementary material for: Emergence of genotype C1 Enterovirus A71 and its link with antigenic variation of virus in Taiwan
Source: PLoS Pathog. 2020 Sep 16;16(9):e1008857. doi: 10.1371/journal.ppat.1008857 (PMC7521691; doi:10.1371/journal.ppat.1008857)
Supplement: S1 Table — (PDF) [file ppat.1008857.s004.pdf]

S1 Table. Demographic and clinical characteristics of enrolled patients with acute EV-A71 infections from 2018 to early 2019.

Subject	Age (years)	Myoclonic jerk	Severe neurological involvement[a]	IVIG usage	Isolated EV-A71 (genotype)
1	3.3	yes	yes	yes	TW-209-2018 (C1)
2	0.2	yes	yes	yes	TW-4002-2018 (C1)
3	3.6	yes	yes	yes	TW-5011-2018 (C1)
4	5.8	-	-	-	TW-6001-2018 (B5)
5	5.6	yes	-	-	TW-6012-2018 (C1)
6	11.2	-	-	-	TW-7001-2018 (C1)
7	11.1	-	-	-	TW-7002-2018 (C1)
8	4.5	yes	yes	yes	TW-7013-2018 (C1)
9	5.8	yes	yes	yes	TW-7019-2018 (C1)
10	4.7	yes	yes	yes	TW-7025-2018 (C1)
11	5.4	yes	yes	yes	TW-7026-2018 (C1)
12	1.4	-	-	-	TW-901-2019 (B5)
13	2.8	yes	yes	yes	NA[b]
14	2.2	-	-	-	TW-112-2019 (C1)
15	0.5	-	-	-	TW-106-2019 (C1)
16	1.1	-	-	-	TW-113-2019 (C1)
17	2.4	yes	-	-	TW-122-2019 (C1)
18	5.2	-	-	-	TW-210-2019 (C1)
19	5.6	yes	-	-	TW-225-2019 (C1)
20	3.5	yes	-	-	TW-3018-2019 (C1)
21	4.8	-	-	-	TW-4015-2019 (C1)

[a] Severe neurological involvement was defined as the presence of encephalitis (such as changes in consciousness, seizure, ataxia, or cranial nerve palsy) and/or autonomic nervous system dysregulation (such as tachycardia, hyperthermia, profuse sweating, or transient hypertension) [1].

[b] Subject 13 was diagnosed with acute EV-A71 infection based on positive reverse-transcriptase polymerase chain reaction of EV-A71 of rectal swab. We failed to isolate EV-A71 from rectal and throat swabs from subject 13.

Abbreviations; IVIG, intravenous immunoglobulin; TW, Taiwan.
