# Supplementary material for: Emergence of genotype C1 Enterovirus A71 and its link with antigenic variation of virus in Taiwan
Source: PLoS Pathog. 2020 Sep 16;16(9):e1008857. doi: 10.1371/journal.ppat.1008857 (PMC7521691; doi:10.1371/journal.ppat.1008857)
Supplement: S2 Table — (DOCX) [file ppat.1008857.s005.docx]

| **S2 Table. The complete VP2-VP3-VP1 sequences of enteroviruses.** | | | | |
| --- | --- | --- | --- | --- |
| Virus | Genotype | Year | Region | Sequence |
| BrCr (GenBank: JN874547) | A | 1970 | USA | SPSAEACGYSDRVAQLTIGNSTITTQEAANIIVGYGEWPSYCSDNDATAVDKPTRPDVSVNRFYTLDTKLWEKSSKGWYWKFPDVLTETGVFGQNAQFHYLYRSGFCIHVQCNASKFHQGALLVAVLPEYVIGTVAGGTGTENSHPPYKQTQPGADGFELQHPYVLDAGIPISQLTVCPHQWINLRTNNCATIIVPYMNTLPFDSALNHCNFGLLVVPISPLDFDQGATPVIPITITLAPMCSEFAGLRQAVTQGFPTELKPGTNQFLTTDDGVSAPILPNFHPTPCIHIPGEVRNLLELCQVETILEVNNVPTNATSLMERLRFPVSAQAGKGELCAVFRADPGRDGPWQSTMLGQLCGYYTQWSGSLEVTFMFTGSFMATGKMLIAYTPPGGPLPKDRATAMLGTHVIWDFGLQSSVTLVIPWISNTHYRAHARDGVFDYYTTGLVSIWYQTNYVVPIGAPNTAYIIALAAAQKNFTMKLCKDTSDILQTATIQGDRVADVIESSIGDSVSKALTQALPAPTGQNTQVSSHRLDTGKVPALQAAEIGASSNASDESMIETRCVLNSHSTAETTLDSFFSRAGLVGEIDLPLKGTTNPNGYANWDIDITGHAQMRRKVELFTYMRFDAEFTFVACTPTGEVVPQLLQYMFVPPGAPKPDSRDSLAWQTATNPSVFVKLSDPPAQVSVPFMSPASAYQWFYDGYPTFGEHKQEKDLEYGACPNNMMGTFSVRTVGSSKSKYPLVIRIYMRMKHVRAWIPRPMRNQNYLFKSNPNYAGNSIKPTGTSRTAITTL |
| 0756-MAA-97 (GenBank: AF135935) | C1 | 1997 | Malaysia | GDRVADVIESSIGDSVSRALTQALPAPTGQNTQVSSHRLDTGKVPALQAAEIGASSNASDESMIETRCVLNSHSTAETTLDSFFSRAGLVGEIDLPLKGTTNPNGYANWDIDITGYAQMRRKVELFTYMRFDAEFTFVACTPTGEVVPQLLQYMFVPPGAPKPDSRESLAWQTATNPSVFVKLSDPPAQVSVPFMSPASAYQWFYDGYPTFGEHKQEKDLEYGACPNNMMGTFSVRTVGTSKSKYPLVIRIYMRMKHVRAWVPRPMRNQNYLFKANPNYAGNSIKPTGTSRTAITTL |
| 1118-MAA-98 (GenBank: AF190576) | C1 | 1998 | Malaysia | GDRVADVIESSIGDSVSRALTQALPAPTGQNTQVSSHRLDTGKVPALQAAEIGASSNASDESMIETRCVLNSHSTAETTLDSFFSRAGLVGEIDLPLEGTTNPNGYANWDIDITGYAQMRRKVELFTYMRFDAEFTFVACTPTGEVVPQLLQYMFVPPGAPKPDSRESLAWQTATNPSVFVKLSDPPAQVSVPFMSPASAYQWFYDGYPTFGEHKQEKDLEYGACPNNMMGTFSVRTVGTSKSKYPLVIRIYMRMKHVRAWVPRPMRNQNYLFKANPNYAGNSIKPTGTSRTAITTL |
| S10862/SAR/98 (GenBank: AF376080) | C1 | 1998 | Malaysia | GDRVADVIESSIGDSVSRALTQALPAPTGQNTQVSSHRLDTGKVPALQAAEIGASSNASDESMIETRCVLNSHSTAETTLDSFFSRAGLVGEIDLPLEGTTNPNGYANWDIDITGYAQMRRKVELFTYMRFDAEFTFVACTPTGEVVPQLLQYMFVPPGAPKPDSRESLAWQTATNPSVFVKLSDPPAQVSVPFMSPASAYQWFYDGYPTFGEHKQEKDLEYGACPNNMMGTFSVRTVGTSKSKYPLVIRIYMRMKHVRAWIPRPMRNQNYLFKANPNYAGNSIKPTGASRTAITTL |
| S11051/SAR/98 (GenBank: AF376081) | C1 | 1998 | Malaysia | GDRVADVIESSIGDSVSRALTQALPAPTGQNTQVSSHRLDTGKVPALQAAEIGASSNASDESMIETRCVLNSHSTAETTLDSFFSRAGLVGEIDLPLKGTTNPNGYANWDIDITGYAQMRRKVELFTYMRFDAEFTFVACTPTGEVVPQLLQYMFVPPGAPKPDSRESLAWQTATNPSVFVKLSDPPAQVSVPFMSPASAYQWFYDGYPTFGEHKQEKDLEYGACPNNMMGTFSVRTVGTSKSKYPLVIRIYMRMKHVRAWIPRPMRNQNYLFKANPNYAGNSIKPTGASRTAITTL |
| TW-4215-1998 (GenBank: JN874553) | C1 | 1998 | Taiwan | SPSAEACGYSDRVAQLTIGNSTITTLEAANIIVGYGEWPSYCSDSDATAVDKPTRPDVSVNRFYTLDTKLWEKSSKGWYWKFPDVLTETGVFGQNAQFHYLYRSGFCIHVQCNASKFHQGALLVAVLPEYVIGTVAGGTGTEDSHPPYKQTQPGADGFELQHPYVLDAGIPISQLTVCPHQWINLRTNNCATIIVPYINALPFDSALNHCNFGLLVVHISPLDFDQGATPVIPITITLAPMCSEFAGLRQAVTQGFPTELKPGTNQFLTTDDGVSAPILPNFHPTPCIHIPGEVRNLLELCQVETILEVNNVPTNATSLMERLRFPVSAQAGKGELCAVFRADPGRNGPWQSTLLGQLCGYYTQWSGSLEVTFMFTGSFMATGKMLIAYTPPGGPLPKDRATAMLGTHVIWDFGLQSSVTLVIPWISNTHYRAHARDGVFDYYTTGLVSIWYQTNYVVPIGAPNTAYIIALAAAQKNFTMKLCKDASDILQTGTIQGDRVADVIESSIGDSVSRALTQALPAPTGQNTQVSSHRLDTGKVPALQAAEIGASSNASDESMIETRCVLNSHSTAETTLDSFFSRAGLVGEIDLPLEGTTNPNGYANWDIDITGYAQMRRKVELFTYMRFDAEFTFVACTPTGQVVPQLLQYMFVPPGAPKPDSRESLAWQTATNPSVFVKLTDPPAQVSVPFMSPASAYQWFYDGYPTFGEHKQEKDLEYGACPNNMMGTFSVRTVGTSKSKYPLVIRIYMRMKHVRAWVPRPMRNQNYLFKANPNYAGNSIKPTGASRTAITTF |
| 1M-Australia-12-00 (GenBank: DQ341361) | C1 | 2000 | Australia | SPSAEACGYSDRVAQLTIGNSTITTQEAANIIVGYGEWPSYCSDSDATAVDKPTRPDVSVNRFYTLDTKLWEKSSKGWYWKFPDVLTETGVFGQNAQFHYLYRSGFCIHVQCNASKFHQGALLVAVLPEYVIGTVAGGTGTEDSHPPYKQTQPGADGFELQHPYVLDAGIPISQLTVCPHQWINLRTNNCATIIVPYINALPFDSALNHCNFGLLVVPISPLDFDQGATPVIPITITLAPMCSEFAGLRQAVTQGFPTELKPGTNQFLTTDDGVSAPILPNFHPTPCIHIPGEVRNLLELCQVETILEVNNVPTNATSLMERLRFPVSAQAGKGELCAVFRADPGRNGPWQSTLLGQLCGYYTQWSGSLEVTFMFTGSFMATGKMLIAYTPPGGPLPKDRATAMLGTHVIWDFGLQSSVTLVIPWISNTHYRAHARDGVFDYYTTGLVSIWYQTNYVVPIGAPNTAYIIALAAAQKNFTMKLCKDASDILQTGTIQGDRVADVIESSIGDSVSRALTQALPAPTGQNTQVSSHRLDTGKVPALQAAEIGASSNASDESMIETRCVLNSHSTAETTLDSFFSRAGLVGEIDLPLEGTTNPNGYANWDIDITGYAQMRRKVELFTYMRFDAEFTFVACTPTGEVVPQLLQYMFVPPGAPKPDSRESLAWQTATNPSVFVKLSDPPAQVSVPFMSPASAYQWFYDGYPTFGEHKQEKDLEYGACPNNMMGTFSVRTVGTSKSKYPLVIRIYMRMKHVRAWIPRPMRNQNYLFKANPNYAGNSIKPTGTSRTAITTL |
| 37507/TH/DE (GenBank: KU641501) | C1 | 2015 | Germany, 4y, meningitis | SPSAEACGYSDRVAQLTIGNSTITTQEAANIIVGYGEWPSYCSDSDATAVDKPTRPDVSVNRFYTLDTKLWEKSSKGWYWKFPDVLTETGVFGQNAQFHYLYRSGFCIHVQCNASKFHQGALLVAVLPEYVIGTVAGGTGTEDSHPPYKQTQPGADGFELQHPYVLDAGVPISQLTVCPHQWINLRTNNCATIIVPYINALPFDSALNHCNFGLLVVPISPLDFDQGATPVIPITITLAPMCSEFAGLRQAVTQGFPTELKPGTNQFLTTDDGVSAPILPNFHPTPCIHIPGEVRNLLELCQVETILEVNNVPTNATSLMERLRFPVSAQAGKGELCAVFRADPGRDGPWQSTLLGQLCGYYTQWSGSLEVTFMFTGSFMATGKMLIAYTPPGGPLPKDRATAMLGTHVIWDFGLQSSVTLVIPWISNTHYRAHARDGVFDYYTTGLVSIWYQTNYVVPIGAPNTAYIIALAAAQKNFTMKLCKDSSDILQTGTIQGDRVADVIESSIGDSMSRALTQALPAPTGQNTQVSSHRLDTGKVPALQAAEIGASSNASDESMIETRCVLNSHSTAETTLDSFFSRAGLVGEIDLPLEGTTNPNGYANWDIDITGYAQMRRKVELFTYMRFDAEFTFVACTPTGEVVPQLLQYMFVPPGAPKPDSRESLAWQTATNPSVFVKLSDPPAQVSVPFMSPASAYQWFYDGYPTFGEHKQEKDLEYGACPNNMMGTFSVRTVGTSKSKYPLVIRIYMRMKHVRAWVPRPMRNQNYLFKANPNYAGNSIKPTGTSRTAITTL |
| 44172/RP/DE (GenBank: KU641507) | C1 | 2015 | Germany, 1y, meningitis | SPSAEACGYSDRVAQLTIGNSTITTQEAANIIVGYGEWPSYCSDSDATAVDKPTRPDVSVNRFYTLDTKLWEKSSKGWYWKFPDVLTETGVFGQNAQFHYLYRSGFCIHVQCNASKFHQGALLVAVLPEYVIGTVAGGTGTEDSHPPYKQTQPGADGFELQHPYVLDAGVPISQLTVCPHQWINLRTNNCATIIVPYINALPFDSALNHCNFGLLVVPISPLDFDQGATPVIPITITLAPMCSEFAGLRQAVTQGFPTELKPGTNQFLTTDDGVSAPILPNFHPTPCVHIPGEVRNLLELCQVETILEVNNVPTNATSLMERLRFPVSAQAGKGELCAVFRADPGRDGPWQSTLLGQLCGYYTQWSGSLEVTFMFTGSFMATGKMLIAYTPPGGPLPKDRATAMLGTHVIWDFGLQSSVTLVIPWISNTHYRAHARDGVFDYYTTGLVSIWYQTNYVVPIGAPNTAYIIALAAAQKNFTMKLCKDSSDILQTGTIQGDRVADVIESSIGDSMSRALTQALPAPTGQNTQVSSHRLDTGKVPALQAAEIGASSNASDESMIETRCVLNSHSTAETTLDSFFSRAGLVGEIDLPLEGTTNPNGYANWDIDITGYAQMRRKVELFTYMRFDAEFTFVACTPTGEVVPQLLQYMFVPPGAPKPDSRESLAWQTATNPSVFVKLSDPPAQVSVPFMSPASAYQWFYDGYPTFGEHKQEKDLEYGACPNNMMGTFSVRTVGTSKSKYPLVIRIYMRMKHVRAWVPRPMRNQNYLFKANPNYAGNSIKPTGTSRTAITTL |
| 44932/BE/DE (GenBank: KU641506) | C1 | 2015 | Germany, 1y, meningitis | SPSAEACGYSDRVAQLTIGNSTITTQEAANIIVGYGEWPSYCSDSDATAVDKPTRPDVSVNRFYTLDTKLWEKSSKGWYWKFPDVLTETGVFGQNAQFHYLYRSGFCIHVQCNASKFHQGALLVAVLPEYVIGTVAGGTGTEDSHPPYKQTQPGADGFELQHPYVLDAGVPISQLTVCPHQWINLRTNNCATIIVPYINALPFDSALNHCNFGLLVVPISPLDFDQGATPVIPITITLAPMCSEFAGLRQAVTQGFPTELKPGTNQFLTTDDGVSAPILPNFHPTPCIHIPGEVRNLLELCQVETILEVNNVPTNATSLMERLRFPVSAQAGKGELCAVFRADPGRDGPWQSTLLGQLCGYYTQWSGSLEVTFMFTGSFMATGKMLIAYTPPGGPLPKDRATAMLGTHVIWDFGLQSSVTLVIPWISNTHYRAHARDGVFDYYTTGLVSIWYQTNYVVPIGAPNTAYIIALAAAQKNFTMKLCKDSSDILQTGTIQGDRVADVIESSIGDSMSRALTQALPAPTGQNTQVSSHRLDTGKVPALQAAEIGASSNASDESMIETRCVLNSHSTAETTLDSFFSRAGLVGEIDLPLEGTTNPNGYANWDIDITGYAQMRRKVELFTYMRFDAEFTFVACTPTGEVVPQLLQYMFVPPGAPKPDSRESLAWQTATNPSVFVKLSDPPAQVSVPFMSPASAYQWFYDGYPTFGEHKQEKDLEYGACPNNMMGTFSVRTVGTSKSKYPLVIRIYMRMKHVRAWVPRPMRNQNYLFKANPNYAGNSIKPTGTSRTAITTL |
| TOU307017\|FRA16 (GenBank: LR027522) | C1 | 2016 | France, neonatal sepsis | SPSAEACGYSDRVAQLTIGNSTITTQEAANIIVGYGEWPSYCSDSDATAVDKPTRPDVSVNRFYTLDTKLWEKSSKGWYWKFPDVLTETGVFGQNAQFHYLYRSGFCIHVQCNASKFHQGALLVAVLPEYVIGTVAGGTGTEDSHPPYKQTQPGADGFELQHPYVLDAGIPISQLTVCPHQWINLRTNNCATIIVPYINALPFDSALNHCNFGLLVVPISPLDFDQGATPVIPITITLAPMCSEFAGLRQAVTQGFPTELKPGTNQFLTTDDGVSAPILPNFHPTPCIHIPGEVRNLLELCQVETILEVNNVPTNATSLMERLRFPVSAQAGKGELCAVFRADPGRDGPWQSTLLGQLCGYYTQWSGSLEVTFMFTGSFMATGKMLIAYTPPGGPLPKDRATAMLGTHVIWDFGLQSSVTLVIPWISNTHYRAHARDGVFDYYTTGLVSIWYQTNYVVPIGAPNTAYIIALAAAQKNFTMKLCKDSSDILQTGTIQGDRVADVIESSIGDSMSRALTQALPAPTGQNTQVSSHRLDTGKVPALQAAEIGASSNASDESMIETRCVLNSHSTAETTLDSFFSRAGLVGEIDLPLEGTTNPNGYANWDIDITGYAQMRRKVELFTYMRFDAEFTFVACTPTGEVVPQLLQYMFVPPGAPKPDSRESLAWQTATNPSVFVKLSDPPAQVSVPFMSPASAYQWFYDGYPTFGEHKQEKDLEYGACPNNMMGTFSVRAVGTSKSKYPLVIRIYMRMKHVRAWVPRPMRNQNYLFKANPNYAGNSIKPTGTSRTAITTL |
| TOU307036\|FRA16 (GenBank: LR027532) | C1 | 2016 | France, 3m, fever | SPSAEACGYSDRVAQLTIGNSTITTQEAANIIVGYGEWPSYCSDSDATAVDKPTRPDVSVNRFYTLDTKLWEKSSKGWYWKFPDVLTETGVFGQNAQFHYLYRSGFCIHVQCNASKFHQGALLVAVLPEYVIGTVAGGTGTEDSHPPYKQTQPGADGFELQHPYVLDAGVPISQLTVCPHQWINLRTNNCATIIVPYINALPFDSALNHCNFGLLVVPISPLDFDQGATPVIPITITLAPMCSEFAGLRQAVTQGFPTELKPGTNQFLTTDDGVSAPILPNFHPTPCIHIPGEVRNLLELCQVETILEVNNVPTNATSLMERLRFPVSAQAGKGELCAVFRADPGRDGPWQSTLLGQLCGYYTQWSGSLEVTFMFTGSFMATGKMLIAYTPPGGPLPKDRATAMLGTHVIWDFGLQSSVTLVIPWISNTHYRAHARDGVFDYYTTGLVSIWYQTNYVVPIGAPNTAYIIALAAAQKNFTMKLCKDSSDILQTGTIQGDRVADVIESSIGDSMSRALTQALPAPTGQNTQVSSHRLDTGKVPALQAAEIGASSNASDESMIETRCVLNSHSTAETTLDSFFSRAGLVGEIDLPLEGTTNPNGYANWDIDITGYAQMRRKVELFTYMRFDAEFTFVACTPTGEVVPQLLQYMFVPPGAPKPDSRESLAWQTATNPSVFVKLSDPPAQVSVPFMSPASAYQWFYDGYPTFGEHKQEKDLEYGACPNNMMGTFSVRTVGTSKSKYPLVIRIYMRMKHVRAWVPRPMRNQNYLFKANPNYAGNSIKPTGTSRTAITTL |
| AMI302001\|FRA16 (GenBank: LR027546) | C1 | 2016 | France, 5y, myelitis | SPSAEACGYSDRVAQLTIGNSTITTQEAANIIVGYGEWPSYCSDSDATAVDKPTRPDVSVNRFYTLDTKLWEKSSKGWYWKFPDVLTETGVFGQNAQFHYLYRSGFCIHVQCNASKFHQGALLVAVLPEYVIGTVAGGTGTEDSHPPYKQTQPGADGFELQHPYVLDAGVPISQLTVCPHQWINLRTNNCATIIVPYINALPFDSALNHCNFGLLVVPISPLDFDQGATPVIPITITLAPMCSEFAGLRQAVTQGFPTELKPGTNQFLTTDDGVSAPILPNFHPTPCIHIPGEVRNLLELCQVETILEVNNVPTNATSLMERLRFPVSAQAGKGELCAVFRADPGRDGPWQSTLLGQLCGYYTQWSGSLEVTFMFTGSFMATGKMLIAYTPPGGPLPKDRATAMLGTHVIWDFGLQSSVTLVIPWISNTHYRAHARDGVFDYYTTGLVSIWYQTNYVVPIGAPNTAYIIALAAAQKNFTMKLCKDSSDILQTGTIQGDRVADVIESSIGDSMSRALTQALPAPTGQNTQVSSHRLDTGKVPALQAAEIGASSNASDESMIETRCVLNSHSTAETTLDSFFSRAGLVGEIDLPLEGTTNPNGYANWDIDITGYAQMRRKVELFTYMRFDAEFTFVACTPTGEVVPQLLQYMFVPPGAPKPDSRESLAWQTATNPSVFVKLSDPPAQVSVPFMSPASAYQWFYDGYPTFGEHKQEKDLEYGACPNNMMGTFSVRTVGTSKSKYPLVIRIYMRMKHVRAWVPRPMRNQNYLFKANPNYAGNSIKPTGTSRTAITTL |
| 31170101 (GenBank: LC321990) | C1 | 2017 | Japan | SPSAEACGYSDRVAQLTIGNSTITTQEAANIIVGYGEWPSYCSDSDATAVDKPTRPDVSVNRFYTLDTKLWEKSSKGWYWKFPDVLTETGVFGQNAQFHYLYRSGFCIHVQCNASKFHQGALLVAVLPEYVIGTVAGGTGTEDSHPPYKQTQPGADGFELQHPYVLDAGVPISQLTVCPHQWINLRTNNCATIIVPYINALPFDSALNHCNFGLLVVPISPLDFDQGATPVIPITITLAPMCSEFAGLRQAVTQGFPTELKPGTNQFLTTDDGVSAPILPNFHPTPCIHIPGEVRNLLELCQVETILEVNNVPTNATSLMERLRFPVSAQAGKGELCAVFRADPGRDGPWQSTLLGQLCGYYTQWSGSLEVTFMFTGSFMATGKMLIAYTPPGGPLPKDRATAMLGTHVIWDFGLQSSVTLVIPWISNTHYRAHARDGVFDYYTTGLVSIWYQTNYVVPIGAPNTAYIIALAAAQKNFTMKLCKDSSDILQTGTIQGDRVADVIESSIGDSMSRALTQALPAPTGQNTQVSSHRLDTGKVPALQAAEIGASSNASDESMIETRCVLNSHSTAETTLDSFFSRAGLVGEIDLPLEGTTNPNGYANWDIDITGYAQMRRKVELFTYMRFDAEFTFVACTPTGEVVPQLLQYMFVPPGAPKPDSRESLAWQTATNPSVFVKLSDPPAQVSVPFMSPASAYQWFYDGYPTFGEHKQEKDLEYGACPNNMMGTFSVRTVGTSKSKYPLVIRIYMRMKHVRAWVPRPMRNQNYLFKANPNYAGNSIKPTGTSRTAITTL |
| 31170112 (GenBank: LC321991) | C1 | 2017 | Japan | SPSAEACGYSDRVAQLTIGNSTITTQEAANIIVGYGEWPSYCSDSDATAVDKPTRPDVSVNRFYTLDTKLWEKSSKGWYWKFPDVLTETGVFGQNAQFHYLYRSGFCIHVQCNASKFHQGALLVAVLPEYVIGTVAGGTGTEDSHPPYKQTQPGADGFELQHPYVLDAGVPISQLTVCPHQWINLRTNNCATIIVPYINALPFDSALNHCNFGLLVVPISPLDFDQGATPVIPITITLAPMCSEFAGLRQAVTQGFPTELKPGTNQFLTTDDGVSAPILPNFHPTPCIHIPGEVRNLLELCQVETILEVNNVPTNATSLMERLRFPVSAQAGKGELCAVFRADPGRDGPWQSTLLGQLCGYYTQWSGSLEVTFMFTGSFMATGKMLIAYTPPGGPLPKDRATAMLGTHVIWDFGLQSSVTLVIPWISNTHYRAHARDGVFDYYTTGLVSIWYQTNYVVPIGAPNTAYIIALAAAQKNFTMKLCKDSSDILQTGTIQGDRVADVIESSIGDSMSRALTQALPAPTGQNTQVSSHRLDTGKVPALQAAEIGASSNASDESMIETRCVLNSHSTAETTLDSFFSRAGLVGEIDLPLEGTTNPNGYANWDIDITGYAQMRRKVELFTYMRFDAEFTFVACTPTGEVVPQLLQYMFVPPGAPKPDSRESLAWQTATNPSVFVKLSDPPAQVSVPFMSPASAYQWFYDGYPTFGEHKQEKDLEYGACPNNMMGTFSVRTVGTSKSKYPLVIRIYMRMKHVRAWVPRPMRNQNYLFKANPNYAGNSIKPTGTSRTAITTL |
| USA/CO/2018-23026 (GenBank: MH718269) | C1 | 2018 | USA | SPSAEACGYSDRVAQLTIGNSTITTQEAANIIVGYGEWPSYCSDSDATAVDKPTRPDVSVNRFYTLDTKLWEKSSKGWYWKFPDVLTETGVFGQNAQFHYLYRSGFCIHVQCNASKFHQGALLVAVLPEYVIGTVAGGTGTEDSHPPYKQTQPGADGFELQHPYVLDAGIPISQLTVCPHQWINLRTNNCATIIVPYINALPFDSALNHCNFGLLVVPISPLDFDQGATPVIPITITLAPMCSEFAGLRQAVTQGFPTELKPGTNQFLTTDDGVSAPILPNFHPTPCIHIPGEVRNLLELCQVETILEVNNVPTNATSLMERLRFPVSAQAGKGELCAVFRADPGRDGPWQSTLLGQLCGYYTQWSGSLEVTFMFTGSFMATGKMLIAYTPPGGPLPKDRATAMLGTHVIWDFGLQSSVTLVIPWISNTHYRAHARDGVFDYYTTGLVSIWYQTNYVVPIGAPNTAYIIALAAAQKNFTMKLCKDSSDILQTGTIQGDRVADVIESSIGDSMSRALTQALPAPTGQNTQVSSHRLDTGKVPALQAAEIGASSNASDESMIETRCVLNSHSTAETTLDSFFSRAGLVGEIDLPLEGTTNPNGYANWDIDITGYAQMRRKVELFTYMRFDAEFTFVACTPTGEVVPQLLQYMFVPPGAPKPDSRESLAWQTATNPSVFVKLSDPPAQVSVPFMSPASAYQWFYDGYPTFGEHKQEKDLEYGACPNNMMGTFSVRTVGTSKSKYPLVIRIYMRMKHVRAWVPRPMRNQNYLFKANPNYAGNSIKPTGTSRTAITTL |
| TW-209-2018 (GenBank: MT747881) | C1 | 2018 | Taiwan | SPSAEACGYSDRVAQLTIGNSTITTQEAANIIVGYGEWPSYCSDSDATAVDKPTRPDVSVNRFYTLDTKLWEKSSKGWYWKFPDVLTETGVFGQNAQFHYLYRSGFCIHVQCNASKFHQGALLVAVLPEYVIGTVAGGTGTEDSHPPYKQTQPGADGFELQHPYVLDAGVPISQLTVCPHQWINLRTNNCATIIVPYINALPFDSALNHCNFGLLVVPISPLDFDQGATPVIPITITLAPMCSEFAGLRQAVTQGFPTELKPGTNQFLTTDDGVSAPILPNFHPTPCIHIPGEVRNLLELCQVETILEVNNVPTNATSLMERLRFPVSAQAGKGELCAVFRADPGRDGPWQSTLLGQLCGYYTQWSGSLEVTFMFTGSFMATGKMLIAYTPPGGPLPKDRATAMLGTHVIWDFGLQSSVTLVIPWISNTHYRAHARDGVFDYYTTGLVSIWYQTNYVVPIGAPNTAYIIALAAAQKNFTMKLCKDSSDILQTGTIQGDRVADVIESSIGDSMSRALTQALPAPTGQNTQVSSHRLDTGKVPALQAAEIGASSNASDESMIETRCVLNSHSTAETTLDSFFSRAGLVGEIDLPLEGTTNPNGYANWDIDITGYAQMRRKVELFTYMRFDAEFTFVACTPTGEVVPQLLQYMFVPPGAPKPDSRESLAWQTATNPSVFVKLSDPPAQVSVPFMSPASAYQWFYDGYPTFGEHKQEKDLEYGACPNNMMGTFSVRTVGTSKSKYPLVIRIYMRMKHVRAWVPRPMRNQNYLFKANPNYAGNSIKPTGTSRTAITTL |
| TW-4002-2018 (GenBank: MT747882) | C1 | 2018 | Taiwan | SPSAEACGYSDRVAQLTIGNSTITTQEAANIIVGYGEWPSYCSDSDATAVDKPTRPDVSVNRFYTLDTKLWEKSSKGWYWKFPDVLTETGVFGQNAQFHYLYRSGFCIHVQCNASKFHQGALLVAVLPEYVIGTVAGGTGTEDSHPPYKQTQPGADGFELQHPYVLDAGVPISQLTVCPHQWINLRTNNCATIIVPYINALPFDSALNHCNFGLLVVPISPLDFDQGATPVIPITITLAPMCSEFAGLRQAVTQGFPTELKPGTNQFLTTDDGVSAPILPNFHPTPCIHIPGEVRNLLELCQVETILEVNNVPTNATSLMERLRFPVSAQAGKGELCAVFRADPGRDGPWQSTLLGQLCGYYTQWSGSLEVTFMFTGSFMATGKMLIAYTPPGGPLPKDRATAMLGTHVIWDFGLQSSVTLVIPWISNTHYRAHARDGVFDYYTTGLVSIWYQTNYVVPIGAPNTAYIIALAAAQKNFTMKLCKDSSDILQTGTIQGDRVADVIESSIGDSMSRALTQALPAPTGQNTQVSSHRLDTGKVPALQAAEIGASSNASDESMIETRCVLNSHSTAETTLDSFFSRAGLVGEIDLPLEGTTNPNGYANWDIDITGYAQMRRKVELFTYMRFDAEFTFVACTPTGEVVPQLLQYMFVPPGAPKPDSRESLAWQTATNPSVFVKLSDPPAQVSVPFMSPASAYQWFYDGYPTFGEHKQEKDLEYGACPNNMMGTFSVRTVGTSKSKYPLVIRIYMRMKHVRAWVPRPMRNQNYLFKANPNYAGNSIKPTGTSRTAITTL |
| TW-5011-2018 (GenBank: MT747883) | C1 | 2018 | Taiwan | SPSAEACGYSDRVAQLTIGNSTITTQEAANIIVGYGEWPSYCSDSDATAVDKPTRPDVSVNRFYTLDTKLWEKSSKGWYWKFPDVLTETGVFGQNAQFHYLYRSGFCIHVQCNASKFHQGALLVAVLPEYVIGTVAGGTGTEDSHPPYKQTQPGADGFELQHPYVLDAGVPISQLTVCPHQWINLRTNNCATIIVPYINALPFDSALNHCNFGLLVVPISPLDFDQGATPVIPITITLAPMCSEFAGLRQAVTQGFPTELKPGTNQFLTTDDGVSAPILPNFHPTPCIHIPGEVRNLLELCQVETILEVNNVPTNATSLMERLRFPVSAQAGKGELCAVFRADPGRGGPWQSTLLGQLCGYYTQWSGSLEVTFMFTGSFMATGKMLIAYTPPGGPLPKDRSTAMLGTHVIWDFGLQSSVTLVIPWISNTHYRAHARDGVFDYYTTGLVSIWYQTNYVVPIGAPNTAYIIALAAAQKNFTMKLCKDSSDILQTGTIQGDRVADVIESSIGDSMSRALTQALPAPTGQNTQVSSHRLDTGKVPALQAAEIGASSNASDESMIETRCVLNSHSTAETTLDSFFSRAGLVGEIDLPLEGTTNPNGYANWDIDITGYAQMRRKVELFTYMRFDAEFTFVACTPTGEVVPQLLQYMFVPPGAPKPDSRESLAWQTATNPSVFVKLSDPPAQVSVPFMSPASAYQWFYDGYPTFGEHKQEKDLEYGACPNNMMGTFSVRTVGTSKSKYPLVIRIYMRMKHVRAWVPRPMRNQNYLFKANPNYAGNSIKPTGTSRTAITTL |
| TW-6012-2018 (GenBank: MT747884) | C1 | 2018 | Taiwan | SPSAEACGYSDRVAQLTIGNSTITTQEAANIIVGYGEWPSYCSDSDATAVDKPTRPDVSVNRFYTLDTKLWEKSSKGWYWKFPDVLTETGVFGQNAQFHYLYRSGFCIHVQCNASKFHQGALLVAVLPEYVIGTVAGGTGTEDSHPPYKQTQPGADGFELQHPYVLDAGVPISQLTVCPHQWINLRTNNCATIIVPYINALPFDSALNHCNFGLLVVPISPLDFDQGATPVIPITITLAPMCSEFAGLRQAVTQGFPTELKPGTNQFLTTDDGVSAPILPNFHPTPCIHIPGEVRNLLELCQVETILEVNNVPTNATSLMERLRFPVSAQAGKGELCAVFRADPGRDGPWQSTLLGQLCGYYTQWSGSLEVTFMFTGSFMATGKMLIAYTPPGGPLPKDRSTAMLGTHVIWDFGLQSSVTLVIPWISNTHYRAHARDGVFDYYTTGLVSIWYQTNYVVPIGAPNTAYIIALAAAQKNFTMKLCKDSSDILQTGTIQGDRVADVIESSIGDSMSRALTQALTAPTGQNTQVSSHRLDTGKVPALQAAEIGASSNASDESMIETRCVLNSHSTAETTLDSFFSRAGLVGEIDLPLEGTTNPNGYANWDIDITGYAQMRRKVELFTYMRFDAEFTFVACTPTGEVVPQLLQYMFVPPGAPKPDSRESLAWQTATNPSVFVKLSDPPAQVSVPFMSPASAYQWFYDGYPTFGEHKQEKDLEYGACPNNMMGTFSVRTVGTSKSKYPLVIRIYMRMKHVRAWVPRPMRNQNYLFKANPNYAGNSIKPTGTSRTAITTL |
| TW-7001-2018 (GenBank: MT747885) | C1 | 2018 | Taiwan | SPSAEACGYSDRVAQLTIGNSTITTQEAANIIVGYGEWPSYCSDSDATAVDKPTRPDVSVNRFYTLDTKLWEKSSKGWYWKFPDVLTETGVFGQNAQFHYLYRSGFCIHVQCNASKFHQGALLVAVLPEYVIGTVAGGTGTEDSHPPYKQTQPGADGFELQHPYVLDAGVPISQLTVCPHQWINLRTNNCATIIVPYINALPFDSALNHCNFGLLVVPISPLDFDQGATPVIPITITLAPMCSEFAGLRQAVTQGFPTELKPGTNQFLTTDDGVSAPILPNFHPTPCIHIPGEVRNLLELCQVETILEVNNVPTNATSLMERLRFPVSAQAGKGELCAVFRADPGRDGPWQSTLLGQLCGYYTQWSGSLEVTFMFTGSFMATGKMLIAYTPPGGPLPKDRSTAMLGTHVIWDFGLQSSVTLVIPWISNTHYRAHARDGVFDYYTTGLVSIWYQTNYVVPIGAPNTAYIIALAAAQKNFTMKLCKDSSDILQTGTIQGDRVADVIESSIGDSMSRALTQALPAPTGQNTQVSSHRLDTGKVPALQAAEIGASSNASDESMIETRCVLNSHSTAETTLDSFFSRAGLVGEIDLPLEGTTNPNGYANWDIDITGYAQMRRKVELFTYMRFDAEFTFVACTPTGEVVPQLLQYMFVPPGAPKPDSRESLAWQTATNPSVFVKLSDPPAQVSVPFMSPASAYQWFYDGYPTFGEHKQEKDLEYGACPNNMMGTFSVRTVGTSKSKYPLVIRIYMRMKHVRAWVPRPMRNQNYLFKANPNYAGNSIKPTGTSRTAITTL |
| TW-7002-2018 (GenBank: MT747886) | C1 | 2018 | Taiwan | SPSAEACGYSDRVAQLTIGNSTITTQEAANIIVGYGEWPSYCSDSDATAVDKPTRPDVSVNRFYTLDTKLWEKSSKGWYWKFPDVLTETGVFGQNAQFHYLYRSGFCIHVQCNASKFHQGALLVAVLPEYVIGTVAGGTGTEDSHPPYKQTQPGADGFELQHPYVLDAGVPISQLTVCPHQWINLRTNNCATIIVPYINALPFDSALNHCNFGLLVVPISPLDFDQGATPVIPITITLAPMCSEFAGLRQAVTQGFPTELKPGTNQFLTTDDGVSAPILPNFHPTPCIHIPGEVRNLLELCQVETILEVNNVPTNATSLMERLRFPVSAQAGKGELCAVFRADPGRDGPWQSTLLGQLCGYYTQWSGSLEVTFMFTGSFMATGKMLIAYTPPGGPLPKDRSTAMLGTHVIWDFGLQSSVTLVIPWISNTHYRAHARDGVFDYYTTGLVSIWYQTNYVVPIGAPNTAYIIALAAAQKNFTMKLCKDSSDILQTGTIQGDRVADVIESSIGDSMSRALTQALPAPTGQNTQVSSHRLDTGKVPALQAAEIGASSNASDESMIETRCVLNSHSTAETTLDSFFSRAGLVGEIDLPLEGTTNPNGYANWDIDITGYAQMRRKVELFTYMRFDAEFTFVACTPTGEVVPQLLQYMFVPPGAPKPDSRESLAWQTATNPSVFVKLSDPPAQVSVPFMSPASAYQWFYDGYPTFGEHKQEKDLEYGACPNNMMGTFSVRTVGTSKSKYPLVIRIYMRMKHVRAWVPRPMRNQNYLFKANPNYAGNSIKPTGTSRTAITTL |
| TW-7013-2018 (GenBank: MT747887) | C1 | 2018 | Taiwan | SPSAEACGYSDRVAQLTIGNSTITTQEAANIIVGYGEWPSYCSDSDATAVDKPTRPDVSVNRFYTLDTKLWEKSSKGWYWKFPDVLTETGVFGQNAQFHYLYRSGFCIHVQCNASKFHQGALLVAVLPEYVIGTVAGGTGTEDSHPPYKQTQPGADGFELQHPYVLDAGVPISQLTVCPHQWINLRTNNCATIIVPYINALPFDSALNHCNFGLLVVPISPLDFDQGATPVIPITITLAPMCSEFAGLRQAVTQGFPTELKPGTNQFLTTDDGVSAPILPNFHPTPCIHIPGEVRNLLELCQVETILEVNNVPTNATSLMERLRFPVSAQAGKGELCAVFRADPGRDGPWQSTLLGQLCGYYTQWSGSLEVTFMFTGSFMATGKMLIAYTPPGGPLPKDRSTAMLGTHVIWDFGLQSSVTLVIPWISNTHYRAHARDGVFDYYTTGLVSIWYQTNYVVPIGAPNTAYIIALAAAQKNFTMKLCKDSSDILQTGTIQGDRVADVIESSIGDSMSRALTQALPAPTGQNTQVSSHRLDTGKVPALQAAEIGASSNASDESMIETRCVLNSHSTAETTLDSFFSRAGLVGEIDLPLEGTTNPNGYANWDIDITGYAQMRRKVELFTYMRFDAEFTFVACTPTGEVVPQLLQYMFVPPGAPKPDSRESLAWQTATNPSVFVKLSDPPAQVSVPFMSPASAYQWFYDGYPTFGEHKQEKDLEYGACPNNMMGTFSVRTVGTSKSKYPLVIRIYMRMKHVRAWVPRPMRNQNYLFKANPNYAGNSIKPTGTSRTAITTL |
| TW-7019-2018 (GenBank: MT747888) | C1 | 2018 | Taiwan | SPSAEACGYSDRVAQLTIGNSTITTQEAANIIVGYGEWPSYCSDSDATAVDKPTRPDVSVNRFYTLDTKLWEKSSKGWYWKFPDVLTETGVFGQNAQFHYLYRSGFCIHVQCNASKFHQGALLVAVLPEYVIGTVAGGTGTEDSHPPYKQTQPGADGFELQHPYVLDAGVPISQLTVCPHQWINLRTNNCATIIVPYINALPFDSALNHCNFGLLVVPISPLDFDQGATPVIPITITLAPMCSEFAGLRQAVTQGFPTELKPGTNQFLTTDDGVSAPILPNFHPTPCIHIPGEVRNLLELCQVETILEVNNVPTNATSLMERLRFPVSAQAGKGELCAVFRADPGRDGPWQSTLLGQLCGYYTQWSGSLEVTFMFTGSFMATGKMLIAYTPPGGPLPKDRSTAMLGTHVIWDFGLQSSVTLVIPWISNTHYRAHARDGVFDYYTTGLVSIWYQTNYVVPIGAPNTAYIIALAAAQKNFTMKLCKDSSDILQTGTIQGDRVADVIESSIGDSMSRALTQALPAPTGQNTQVSSHRLDTGKVPALQAAEIGASSNASDESMIETRCVLNSHSTAETTLDSFFSRAGLVGEIDLPLEGTTNPNGYANWDIDITGYAQMRRKVELFTYMRFDAEFTFVACTPTGEVVPQLLQYMFVPPGAPKPDSRESLAWQTATNPSVFVKLSDPPAQVSVPFMSPASAYQWFYDGYPTFGEHKQEKDLEYGACPNNMMGTFSVRTVGTSKSKYPLVIRIYMRMKHVRAWVPRPMRNQNYLFKANPNYAGNSIKPTGTSRTAITTL |
| TW-7025-2018 (GenBank: MT747889) | C1 | 2018 | Taiwan | SPSAEACGYSDRVAQLTIGNSTITTQEAANIIVGYGEWPSYCSDSDATAVDKPTRPDVSVNRFYTLDTKLWEKSSKGWYWKFPDVLTETGVFGQNAQFHYLYRSGFCIHVQCNASKFHQGALLVAVLPEYVIGTVAGGTGTEDSHPPYKQTQPGADGFELQHPYVLDAGVPISQLTVCPHQWINLRTNNCATIIVPYINALPFDSALNHCNFGLLVVPISPLDFDQGATPVIPITITLAPMCSEFAGLRQAVTQGFPTELKPGTNQFLTTDDGVSAPILPNFHPTPCIHIPGEVRNLLELCQVETILEVNNVPTNATSLMERLRFPVSAQAGKGELCAVFRADPGRDGPWQSTLLGQLCGYYTQWSGSLEVTFMFTGSFMATGKMLIAYTPPGGPLPKDRSTAMLGTHVIWDFGLQSSVTLVIPWISNTHYRAHARDGVFDYYTTGLVSIWYQTNYVVPIGAPNTAYIIALAAAQKNFTMKLCKDSSDILQTGTIQGDRVADVIESSIGDSMSRALTQALPAPTGQNTQVSSHRLDTGKVPALQAAEIGASSNASDESMIETRCVLNSHSTAETTLDSFFSRAGLVGEIDLPLEGTTNPNGYANWDIDITGYAQMRRKVELFTYMRFDAEFTFVACTPTGEVVPQLLQYMFVPPGAPKPDSRESLAWQTATNPSVFVKLSDPPAQVSVPFMSPASAYQWFYDGYPTFGEHKQEKDLEYGACPNNMMGTFSVRTVGTSKSKYPLVIRIYMRMKHVRAWVPRPMRNQNYLFKANPNYAGNSIKPTGTSRTAITTL |
| TW-7026-2018 (GenBank: MT747890) | C1 | 2018 | Taiwan | SPSAEACGYSDRVAQLTIGNSTITTQEAANIIVGYGEWPSYCSDSDATAVDKPTRPDVSVNRFYTLDTKLWEKSSKGWYWKFPDVLTETGVFGQNAQFHYLYRSGFCIHVQCNASKFHQGALLVAVLPEYVIGTVAGGTGTEDSHPPYKQTQPGADGFELQHPYVLDAGVPISQLTVCPHQWINLRTNNCATVIVPYINALPFDSALNHCNFGLLVVPISPLDFDQGATPVIPITITLAPMCSEFAGLRQAVTQGFPTELKPGTNQFLTTDDGVSAPILPNFHPTPCIHIPGEVRNLLELCQVETILEVNNVPTNATSLMERLRFPVSAQAGKGELCAVFRADPGRDGPWQSTLLGQLCGYYTQWSGSLEVTFMFTGSFMATGKMLIAYTPPGGPLPKDRSTAMLGTHVIWDFGLQSSVTLVIPWISNTHYRAHARDGVFDYYTTGLVSIWYQTNYVVPIGAPNTAYIIALAAAQKNFTMKLCKDSSDILQTGTIQGDRVADVIESSIGDSMSRALTQALPAPTGQNTQVSSHRLDTGKVPALQAAEIGASSNASDESMIETRCVLNSHSTAETTLDSFFSRAGLVGEIDLPLEGTTNPNGYANWDIDITGYAQMRRKVELFTYMRFDAEFTFVACTPTGEVVPQLLQYMFVPPGAPKPDSRESLAWQTATNPSVFVKLSDPPAQVSVPFMSPASAYQWFYDGYPTFGEHKQEKDLEYGACPNNMMGTFSVRTVGTSKSKYPLVIRIYMRMKHVRAWVPRPMRNQNYLFKANPNYAGNSIKPTGTSRTAITTL |
| TW-112-2019 (GenBank: MT747891) | C1 | 2019 | Taiwan | SPSAEACGYSDRVAQLTIGNSTITTQEAANIIVGYGEWPSYCSDSDATAVDKPTRPDVSVNRFYTLDTKLWEKSSKGWYWKFPDVLTETGVFGQNAQFHYLYRSGFCIHVQCNASKFHQGALLVAVLPEYVIGTVAGGTGTEDSHPPYKQTQPGADGFELQHPYVLDAGVPISQLTVCPHQWINLRTNNCATIIVPYINALPFDSALNHCNFGLLVVPISPLDFDQGATPVIPITITLAPMCSEFAGLRQAVTQGFPTELKPGTNQFLTTDDGVSAPILPNFHPTPCIHIPGEVRNLLELCQVETILEVNNVPTNATSLMERLRFPVSAQAGKGELCAVFRADPGRDGPWQSTLLGQLCGYYTQWSGSLEVTFMFTGSFMATGKMLIAYTPPGGPLPKDRATAMLGTHVIWDFGLQSSVTLVIPWISNTHYRAHARDGVFDYYTTGLVSIWYQTNYVVPIGAPNTAYIIALAAAQKNFTMKLCKDSSDILQTGTIQGDRVADVIESSVGDSVSRALTQALPAPTGQNTQVSSHRLDTGKVPALQAAEIGASSNASDESMIETRCVLNSHSTAETTLDSFFSRAGLVGEIDLPLEGTTNPNGYANWDIDITGYAQMRRKVELFTYMRFDAEFTFVACTPTGEVVPQLLQYMFVPPGAPKPDSRESLAWQTATNPSVFVKLSDPPAQVSVPFMSPASAYQWFYDGYPTFGEHKQEKDLEYGACPNNMMGTFSVRTVGTSKSKYPLVIRIYMRMKHVRAWVPRPMRNQNYLFKANPNYAGNSIKPTGTSRTAITTL |
| TW-106-2019 (GenBank: MT747892) | C1 | 2019 | Taiwan | SPSAEACGYSDRVAQLTIGNSTITTQEAANIIVGYGEWPSYCSDSDATAVDKPTRPDVSVNRFYTLDTKLWEKSSKGWYWKFPDVLTETGVFGQNAQFHYLYRSGFCIHVQCNASKFHQGALLVAVLPEYVIGTVAGGTGTEDSHPPYKQTQPGADGFELQHPYVLDAGVPISQLTVCPHQWINLRTNNCATIIVPYINALPFDSALNHCNFGLLVVPISPLDFDQGATPVIPITITLAPMCSEFAGLRQAVTQGFPTELKPGTNQFLTTDDGVSAPILPNFHPTPCIHIPGEVRNLLELCQVETILEVNNVPTNATSLMERLRFPVSAQAGKGELCAVFRADPGRDGPWQSTLLGQLCGYYTQWSGSLEVTFMFTGSFMATGKMLIAYTPPGGPLPKDRATAMLGTHVIWDFGLQSSVTLVIPWISNTHYRAHARDGVFDYYTTGLVSIWYQTNYVVPIGAPNTAYIIALAAAQKNFTMKLCKDSSDILQTGTIQGDRVADVIESSIGDSVSRALTQALPAPTGQNTQVSSHRLDTGKVPALQAAEIGASSNASDESMIETRCVLNSHSTAETTLDSFFSRAGLVGEIDLPLEGTTNPNGYANWDIDITGYAQMRRKVELFTYMRFDAEFTFVACTPTGEVVPQLLQYMFVPPGAPKPDSRESLAWQTATNPSVFVKLSDPPAQVSVPFMSPASAYQWFYDGYPTFGEHKQEKDLEYGACPNNMMGTFSVRTVGTSKSKYPLVIRIYMRMKHVRAWVPRPMRNQNYLFKANPNYAGNSIKPTGTSRTAITTL |
| TW-113-2019 (GenBank: MT747893) | C1 | 2019 | Taiwan | SPSAEACGYSDRVAQLTIGNSTITTQEAANIIVGYGEWPSYCSDSDATAVDKPTRPDVSVNRFYTLDTKLWEKSSKGWYWKFPDVLTETGVFGQNAQFHYLYRSGFCIHVQCNASKFHQGALLVAVLPEYVIGTVAGGTGTEDSHPPYKQTQPGADGFELQHPYVLDAGVPISQLTVCPHQWINLRTNNCATIIVPYINALPFDSALNHCNFGLLVVPISPLDFDQGATPVIPITITLAPMCSEFAGLRQAVTQGFPTELKPGTNQFLTTDDGVSAPILPNFHPTPCIHIPGEVRNLLELCQVETILEVNNVPTNATSLMERLRFPVSAQAGKGELCAVFRADPGRDGPWQSTLLGQLCGYYTQWSGSLEVTFMFTGSFMATGKMLIAYTPPGGPLPKDRATAMLGTHVIWDFGLQSSVTLVIPWISNTHYRAHARDGVFDYYTTGLVSIWYQTNYVVPIGAPNTAYIIALAAAQKNFTMKLCKDSSDILQTGTIQGDRVADVIESSIGDSVSRALTQALPAPTGQNTQVSSHRLDTGKVPALQAAEIGASSNASDESMIETRCVLNSHSTAETTLDSFFSRAGLVGEIDLPLEGTTNPNGYANWDIDITGYAQMRRKVELFTYMRFDAEFTFVACTPTGEVVPQLLQYMFVPPGAPKPDSRESLAWQTATNPSVFVKLSDPPAQVSVPFMSPASAYQWFYDGYPTFGEHKQEKDLEYGACPNNMMGTFSVRTVGTSKSKYPLVIRIYMRMKHVRAWVPRPMRNQNYLFKANPNYAGNSIKPTGTSRTAITTL |
| TW-122-2019 (GenBank: MT747894) | C1 | 2019 | Taiwan | SPSAEACGYSDRVAQLTIGNSTITTQEAANIIVGYGEWPSYCSDSDATAVDKPTRPDVSVNRFYTLDTKLWEKSSKGWYWKFPDVLTETGVFGQNAQFHYLYRSGFCIHVQCNASKFHQGALLVAVLPEYVIGTVAGGTGTEDSHPPYKQTQPGADGFELQHPYVLDAGVPISQLTVCPHQWINLRTNNCATIIVPYINALPFDSALNHCNFGLLVVPISPLDFDQGATPVIPITITLAPMCSEFAGLRQAVTQGFPTELKPGTNQFLTTDDGVSAPILPNFHPTPCIHIPGEVRNLLELCQVETILEVNNVPTNATSLMERLRFPVSAQAGKGELCAVFRADPGRDGPWQSTLLGQLCGYYTQWSGSLEVTFMFTGSFMATGKMLIAYTPPGGPLPKDRATAMLGTHVIWDFGLQSSVTLVIPWISNTHYRAHARDGVFDYYTTGLVSIWYQTNYVVPIGAPNTAYIIALAAAQKNFTMKLCKDSSDILQTGTIQGDRVADVIESSIGDSVSRALTQALPAPTGQNTQVSSHRLDTGKVPALQAAEIGASSNASDESMIETRCVLNSHSTAETTLDSFFSRAGLVGEIDLPLEGTTNPNGYANWDIDITGYAQMRRKVELFTYMRFDAEFTFVACTPTGEVVPQLLQYMFVPPGAPKPDSRESLAWQTATNPSVFVKLSDPPAQVSVPFMSPASAYQWFYDGYPTFGEHKQEKDLEYGACPNNMMGTFSVRTVGTSKSKYPLVIRIYMRMKHVRAWVPRPMRNQNYLFKANPNYAGNSIKPTGTSRTAITTL |
| TW-210-2019 (GenBank: MT747895) | C1 | 2019 | Taiwan | SPSAEACGYSDRVAQLTIGNSTITTQEAANIIVGYGEWPSYCSDSDATAVDKPTRPDVSVNRFYTLDTKLWEKSSKGWYWKFPDVLTETGVFGQNAQFHYLYRSGFCIHVQCNASKFHQGALLVAVLPEYVIGTVAGGTGTEDSHPPYKQTQPGADGFELQHPYVLDAGVPISQLTVCPHQWINLRTNNCATIIVPYINALPFDSALNHCNFGLLVVPISPLDFDQGATPVIPITITLAPMCSEFAGLRQAVTQGFPTELKPGTNQFLTTDDGVSAPILPNFHPTPCIHIPGEVRNLLELCQVETILEVNNVPTNATSLMERLRFPVSAQAGKGELCAVFRADPGRDGPWQSTLLGQLCGYYTQWSGSLEVTFMFTGSFMATGKMLIAYTPPGGPLPKDRATAMLGTHVIWDFGLQSSVTLVIPWISNTHYRAHARDGVFDYYTTGLVSIWYQTNYVVPIGAPNTAYIIALAAAQKNFTMKLCKDSSDILQTGTIQGDRVADVIESSVGDSVSRALTQALPAPTGQNTQVSSHRLDTGKVPALQAAEIGASSNASDESMIETRCVLNSHSTAETTLDSFFSRAGLVGEIDLPLEGTTNPNGYANWDIDITGYAQMRRKVELFTYMRFDAEFTFVACTPTGEVVPQLLQYMFVPPGAPKPDSRESLAWQTATNPSVFVKLSDPPAQVSVPFMSPASAYQWFYDGYPTFGEHKQEKDLEYGACPNNMMGTFSVRTVGTSKSKYPLVIRIYMRMKHVRAWVPRPMRNQNYLFKANPNYAGNSIKPTGTSRTAITTL |
| TW-225-2019 (GenBank: MT747896) | C1 | 2019 | Taiwan | SPSAEACGYSDRVAQLTIGNSTITTQEAANIIVGYGEWPSYCSDSDATAVDKPTRPDVSVNRFYTLDTKLWEKSSKGWYWKFPDVLTETGVFGQNAQFHYLYRSGFCIHVQCNASKFHQGALLVAVLPEYVIGTVAGGTGTEDSHPPYKQTQPGADGFELQHPYVLDAGVPISQLTVCPHQWINLRTNNCATIIVPYINALPFDSALNHCNFGLLVVPISPLDFDQGATPVIPITITLAPMCSEFAGLRQAVTQGFPTELKPGTNQFLTTDDGVSAPILPNFHPTPCIHIPGEVRNLLELCQVETILEVNNVPTNATSLMERLRFPVSAQAGKGELCAVFRADPGRDGPWQSTLLGQLCGYYTQWSGSLEVTFMFTGSFMATGKMLIAYTPPGGPLPKDRATAMLGTHVIWDFGLQSSVTLVIPWISNTHYRAHARDGVFDYYTTGLVSIWYQTNYVVPIGAPNTAYIIALAAAQKNFTMKLCKDSSDILQTGTIQGDRVADVIESSIGDSVSRALTQALPAPTGQNTQVSSHRLDTGKVPALQAAEIGASSNASDESMIETRCVLNSHSTAETTLDSFFSRAGLVGEIDLPLEGTTNPNGYANWDIDITGYAQMRRKVELFTYMRFDAEFTFVACTPTGEVVPQLLQYMFVPPGAPKPDSRESLAWQTATNPSVFVKLSDPPAQVSVPFMSPASAYQWFYDGYPTFGEHKQEKDLEYGACPNNMMGTFSVRTVGTSKSKYPLVIRIYMRMKHVRAWVPRPMRNQNYLFKANPNYAGNSIKPTGTSRTAITTL |
| TW-3018-2019 (GenBank: MT747897) | C1 | 2019 | Taiwan | SPSAEACGYSDRVAQLTIGNSTITTQEAANIIVGYGEWPSYCSDSDATAVDKPTRPDVSVNRFYTLDTKLWEKSSKGWYWKFPDVLTETGVFGQNAQFHYLYRSGFCIHVQCNASKFHQGALLVAVLPEYVIGTVAGGTGTEDSHPPYKQTQPGADGFELQHPYVLDAGVPISQLTVCPHQWINLRTNNCATIIVPYINALPFDSALNHCNFGLLVVPISPLDFDQGATPVIPITITLAPMCSEFAGLRQAVTQGFPTELKPGTNQFLTTDDGVSAPILPNFHPTPCIHIPGEVRNLLELCQVETILEVNNVPTNATSLMERLRFPVSAQAGKGELCAVFRADPGRDGPWQSTLLGQLCGYYTQWSGSLEVTFMFTGSFMATGKMLIAYTPPGGPLPKDRATAMLGTHVIWDFGLQSSVTLVIPWISNTHYRAHARDGVFDYYTTGLVSIWYQTNYVVPIGAPNTAYIIALAAAQKNFTMKLCKDSSDILQTGTIQGDRVADVIESSIGDSVSRALTQALPAPTGQNTQVSSHRLDTGKVPALQAAEIGASSNASDESMIETRCVLNSHSTAETTLDSFFSRAGLVGEIDLPLEGTTNPNGYANWDIDITGYAQMRRKVELFTYMRFDAEFTFVACTPTGEVVPQLLQYMFVPPGAPKPDSRESLAWQTATNPSVFVKLSDPPAQVSVPFMSPASAYQWFYDGYPTFGEHKQEKDLEYGACPNNMMGTFSVRTVGTSKSKYPLVIRIYMRMKHVRAWVPRPMRNQNYLFKANPNYAGNSIKPTGTSRTAITTL |
| TW-4015-2019 (GenBank: MT747898) | C1 | 2019 | Taiwan | SPSAEACGYSDRVAQLTIGNSTITTQEAANIIVGYGEWPSYCSDSDATAVDKPTRPDVSVNRFYTLDTKLWEKSSKGWYWKFPDVLTETGVFGQNAQFHYLYRSGFCIHVQCNASKFHQGALLVAVLPEYVIGTVAGGTGTEDSHPPYKQTQPGADGFELQHPYVLDAGVPISQLTVCPHQWINLRTNNCATIIVPYINALPFDSALNHCNFGLLVVPISPLDFDQGATPVIPITITLAPMCSEFAGLRQAVTQGFPTELKPGTNQFLTTDDGVSAPILPNFHPTPCIHIPGEVRNLLELCQVETILEVNNVPTNATSLMERLRFPVSAQAGKGELCAVFRADPGRDGPWQSTLLGQLCGYYTQWSGSLEVTFMFTGSFMATGKMLIAYTPPGGPLPKDRATAMLGTHVIWDFGLQSSVTLVIPWISNTHYRAHARDGVFDYYTTGLVSIWYQTNYVVPIGAPNTAYIIALAAAQKNFTMKLCKDSSDILQTGTIQGDRVADVIESSIGDSVSRALTQALPAPTGQNTQVSSHRLDTGKVPALQAAEIGASSNASDESMIETRCVLNSHSTAETTLDSFFSRAGLVGEIDLPLEGTTNPNGYANWDIDITGYAQMRRKVELFTYMRFDAEFTFVACTPTGEVVPQLLQYMFVPPGAPKPDSRESLAWQTATNPSVFVKLSDPPAQVSVPFMSPASAYQWFYDGYPTFGEHKQEKDLEYGACPNNMMGTFSVRTVGTSKSKYPLVIRIYMRMKHVRAWVPRPMRNQNYLFKANPNYAGNSIKPTGTSRTAITTL |
| TW-1691-1999 (GenBank: MT747899) | C2 | 1999 | Taiwan | SPSAEACGYSDRVAQLTIGNSTITTQEAANIIVGYGEWPSYCSDSDATAVDKPTRPDVSVNRFYTLDTKLWEKSSKGWYWKFPDVLTETGVFGQNAQFHYLYRSGFCIHVQCNASKFHQGALLVAVLPEYVIGTVAGGTGTEDSHPPYKQTQPGADGFELQHPYVLDAGIPISQLTVCPHQWINLRTNNCATIIVPYINALPFDSALNHCNFGLLVVPISPLDYDQGATPVIPITITLAPMCSEFAGLRQAVTQGFPTELKPGTNQFLTTDDGVSAPILPNFHPTPCIHIPGEVRNLLELCQVETILEVNNVPTNAASLMERLRFPVSAQAGKGELCAVFRADPGRSGPWQSTLLGQLCGYYTQWSGSLEVTFMFTGSFMATGKMLIAYTPPGGPLPKDRATAMLGTHVIWDFGLQSSVTLVIPWISNTHYRAHARDGVFDYYTTGLVSIWYQTNYVVPIGAPNTAYIIALAAAQKNFTMKLCKDASDILQTGTIQGDRVADVIESSIGDSVSRALTRALPAPTGQDTQVSSHRLDTGKVPALQAAEIGASSNASDESMIETRCVLNSHSTAETTLDSFFSRAGLVGEIDLPLEGTTNPNGYANWDIDITGYAQMRRKVELFTYMRFDAEFTFVACTPTGEVVPQLLQYMFVPPGAPKPDSRESLAWQTATNPSVFVKLSDPPAQVSVPFMSPASAYQWFYDGYPTFGEHKQEKDLEYGACPNNMMGTFSVRTVGTSKSKYPLVIRIYMRMKHVRAWIPRPMRNQNYLFKANPNYAGNSIKPTGASRTAITTL |
| 03-Korea-00 (GenBank: DQ341356) | C3 | 2000 | Korea | SPSAEACGYSDRVAQLTIGNSTITTQEAANIIVGYGEWPSYCSDSDATAVDKPTRPDVSVNRFYTLDTKLWEKSSKGWYWKFPDVLTETGVFGQNAQFHYLYRSGFCIHVQCNASKFHQGALLVAVLPEYVIGTVAGGTGTEDSHPPYKQTQPGADGFELQHPYVLDAGIPISQLTVCPHQWINLRTNNCATIIVPYINALPFDSALNHCNFGLLVVPISPLDFDQGATPVIPITITLAPMCSEFAGLRQAVTQGFPTELKPGTNQFLTTDDGVSAPILPNFHPTPCIHIPGEVRNLLELCQVETILEVNNVPTNATSLMERLRFPVSAQAGKGELCAVFRADPGRSGPWQSTLLGQLCGYYTQWSGSLEVTFMFTGSFMATGKMLIAYTPPGGPLPKDRATAMLGTHVIWDFGLQSSVTLVIPWISNTHYRAHARDGVFDYYTTGLVSIWYQTNYVVPIGAPNTAYIIALAAAQKNFTMKLCKDASDILQTGTIQGDRVADVIESSIGDSVSRALTQALPAPTGQNTQVSSHRLDTGKVPALQAAEIGASSNASDESMIETRCVLNSHSTAETTLDSFFSRAGLVGEIDLPLEGTTNPNGYANWDIDITGYAQMRRKVELFTYMRFDAEFTFVACTPTGEVVPQLLQYMFVPPGAPKPDSRESLAWQTATNPSVFVKLSDPPAQVSVPFMSPASAYQWFYDGYPTFGEHKQEKDLEYGACPNNMMGTFSVRTVGTSKSKYPLVIRIYMRMKHVRAWVPRPMRNQNYLFKANPNYAGNSIKPTGASRTAITTL |
| TW-96023-2011 (GenBank: KX267855) | C4 | 2011 | Taiwan | SPSAEACGYSDRVAQLTIGNSTITTQEAANIIVGYGEWPSYCSDSDATAVDKPTRPDVSVNRFYTLDTKLWEKSSKGWYWKFPDVLTETGVFGQNAQFHYLYRSGFCIHVQCNASKFHQGALLVAVLPEYVIGTVAGGTGTEDTHPPYKQTQPGADGFELQHPYVLDAGIPISQLTVCPHQWINLRTNNCATIIVPYINALPFDSALNHCNFGLLVVPISPLDYDQGATPVIPITITLAPMCSEFAGLRQAVTQGFPTELKPGTNQFLTTDDGVSAPILPNFHPTPCIHIPGEVRNLLELCQVETILEVNNVPTNATSLMERLRFPVSAQAGKGELCAVFRADPGRNGPWQSTLLGQLCGYYTQWSGSLEVTFMFTGSFMATGKMLIAYTPPGGPLPKDRATAMLGTHVIWDFGLQSSVTLVIPWISNTHYRAHARDGVFDYYTTGLVSIWYQTNYVVPIGAPNTAYIIALAAAQKNFTMKLCKDASDILQTGTIQGDRVADVIESSIGDSVSRALTHALPAPTGQNTQVSSHRLDTGKVPALQAAEIGASSNASDESMIETRCVLNSHSTAETTLDSFFSRAGLVGEIDLPLEGTTNPNGYANWDIDITGYAQMRRKVELFTYMRFDAEFTFVACTPTGQVVPQLLQYMFVPPGAPKPDSRESLAWQTATNPSVFVKLSDPPAQVSVPFMSPASAYQWFYDGYPTFGEHKQEKDLEYGACPNNMMGTFSVRTVGTSKSKYPLVVRIYMRMKHVRAWIPRPMRNQNYLFKANPNYAGNSIKPTGASRTAITTL |
| TW-50555-2016 (GenBank: MT747900) | C4 | 2016 | Taiwan | SPSAEACGYSDRVAQLTIGNSTITTQEAANIIVGYGEWPSYCSDSDATAVDKPTRPDVSVNRFYTLDTKLWEKSSKGWYWKFPDVLTETGVFGQNAQFHYLYRSGFCIHVQCNASKFHQGALLVAVLPEYVIGTVAGGTGTEDTHPPYKQTQPGADGFELQHPYVLDAGIPISQLTVCPHQWINLRTNNCATIIVPYINALPFDSALNHCNFGLLVVPVSPLDYDQGATPVIPITITLAPMCSEFAGLRQAVTQGFPTEPKPGTNQFLTTDDGVSAPILPNFHPTPCIHIPGEVRNLLELCQVETILEVNNVPTNATSLMERLRFPVSAQAGKGELCAVFRADPGRNGPWQSTLLGQLCGYYTQWSGSLEVTFMFTGSFMATGKMLIAYTPPGGPLPKDRATAMLGTHVIWDFGLQSSVTLVIPWISNTHYRAHARDGVFDYYTTGLVSIWYQTNYVVPIGAPNTAYIIALAAAQKNFTMKLCKDASDILQTGTIQGDRVADVIESSIGDSVSRALTHALPAPTGQNTQVSSHRLDTGKVPALQAAEIGASSNASDESMIETRCVLNSHSTAETTLDSFFSRAGLVGEIDLPLEGTTNPNGYANWDIDITGYAQMRRKVELFTYMRFDAEFTFVACTPTGQVVPQLLQYMFVPPGAPKPDSRESLAWQTATNPSVFVKLSDPPAQVSVPFMSPASAYQWFYDGYPTFGEHKQEKDLEYGACPNNMMGTFSVRTVGTSKSKYPLVVRIYMRMKHVRAWIPRPMRNQNYLFKANPNYAGNTIKPTGASRTSITTL |
| TW-1575-2007 (GenBank: JN874558) | C5 | 2007 | Taiwan | SPSAEACGYSDRVAQLTIGNSTITTQEAANIIVGYGEWPSYCSDSDATAVDKPTRPDVSVNRFYTLDTKLWEKSSKGWYWKFPDVLTETGVFGQNAQFHYLYRSGFCIHVQCNASKFHQGALLVAVLPEYVIGTVAGGTGTEDSHPPYKQTQPGAEGFELQHPYVLDAGIPISQLTVCPHQWINLRTNNCATIIVPYINACPFDFALNHCNFGLLVVPISPLDFDQGATPVIPITITLAPMCSEFAGLRQAVTQGFPTELKPGTNQFLTTDDGVSAPILPNFHPTPCIHIPGEVRNLLELCQVETILEVNNVPTNATSLMERLRFPVSAQAGKGELCAVFRADPGRSGPWQSTLLGQLCGYYTQWSGSLEVTFMFTGSFMATGKMLIAYTPPGGPLPKDRATAMLGTHVIWDFGLQSSVTLVIPWISNTHYRAHARDGVFDYYTTGLVSIWYQTNYVVPIGAPNTAYIIALAAAQKNFTMKLCKDASDILQTGTIQGDRVADVIESSIGDSVSRALTQALPAPTGQNTQVSSHRLDTGKVPALQAAEIGASSNASDESMIETRCVLNSHSTAETTLDSFFSRAGLVGEIDLPLEGTTNPNGYANWDIDITGYAQMRRKVELFTYMRFDAEFTFVACTPTGEVVPQLLQYMFVPPGAPKPDSRESLAWQTATNPSVFVKLSDPPAQVSVPFMSPASAYQWFYDGYPTFGEHKQEKDLEYGACPNNMMGTFSVRTVGTSKSKYPLVIRIYMRMKHVRAWVPRPMRNQNYLFKANPNYAGNSIKPTGASRTAITTL |
| TW-237-1986 (GenBank: FJ357380) | B1 | 1986 | Taiwan | SPTAEACGYSDRVAQLTIGNSTITTQEAANIIVGYGEWPSYCSDDDATAVDKPTRPDVSVNRFYTLDTKLWEKSSKGWYWKFPDVLTETGVFGQNAQFHYLYRSGFCIHVQCNASKFHQGALLVAILPEYVIGTVAGGTGTEDSHPPYKQTQPGADGFELQHPYVLDAGIPISQLTVRPHQWINLRTNNCATIIVPYMNTLPFDSALNHCNFGLLVVPISPLDFDQGATPVIPITITLAPMCSEFAGLRQAVTQGFPTELKPGTNQFLTTDDGVSAPILPNFHPTPCIHIPGEVRNLLELCQVETILEVNNVPTNATSLMERLRFPVSAQAGKGELCAVFRADPGRDGPWQSTMLGQLCGYYTQWSGSLEVTFMFTGSFMATGKMLIAYTPPGGPLPKDRATAMLGTHVIWDFGLQSSVTLVIPWISNTHYRAHARDGVFDYYTTGLVSIWYQTNYVVPIGAPNTAYIIALAAAQKNFTMKLCKDTSHILQTASIQGDRVADVIESSIGDSMSRALTQALPAPTGQNTQVSSHRLDTGEVPALQAAEIGASSNTSDESMIETRCVLNSHSTAETTLDSFFSRAGLVGEIDLPLKGTTNPNGYANWDIDITGYAQMRRKVELFTYMRFDAEFTFVACTPSGEVVPQLFQYMFVPPGAPKPDSRESLAWQTATNPSVFVKLTDPPAQVSVPFMSPASAYQWFYDGYPTFGEHKQEKDLEYGACPNNMMGTFSVRTVGSSKSKYPLVIRIYMRMKHVRAWIPRPMRNQNYLFKANPNYAGNSIKPTGTSRKAITTL |
| MS-7423-87 (GenBank: U22522) | B2 | 1987 | USA | SPSAEACGYSDRVAQLTIGNSTITTQEAANIIVGYGEWPSYCSDDDATAVDKPTRPDVSVNRFYTLDTKLWEKSSKGWYWKFPDVLTETGVFGQNAQFHYLYRSGFCIHVQCNASKFHQGALLVAILPEYVIGTVAGGTGTEDSHPPYKQTQPGADGFELQHPYVLDAGIPISQLTVCPHQWINLRTNNCATIIVPYMNTLPFDSALNHCNFGLLVVPISPLDFDQGATPVIPITITLAPMCSEFGGLRQAVTQGFPTELKPGTNQFLTTDDGVSAPILPNFHPTPCIHIPGEVRNLLELCQVETILEVNNVPTNATSLMERLRFPVSAQAGKGELCAVFRADPGRDGPWQSTMLGQLCGYYTQWSGSLEVTFMFTGSFMATGKMLIAYTPPGGPLPKDRATAMLGTHVIWDFGLQSSVTLVIPWISNTHYRAHARDGVFDYYTTGLVSIWYQTNYVVPIGAPNTAYILALAAAQKNFTMKLCKDTSHILQTASIQGDRVADVIESSIGDSVSRALTQALPAPTGQNTQVSSHRLDTGEVPALQAAEIGASSNTSDESMIETRCVLNSHSTAETTLDSFFSRAGLVGEIDLPLEGTTNPNGYANWDIDITGYAQMRRKVELFTYMRFDAEFTFVACTPTGEVVPQLLQYMFVPPGAPKPESRESLAWQTATNPSVFVKLTDPPAQVSVPFMSPASAYQWFYDGYPTFGEHKQEKDLEYGACPNNMMGTFSVRTVGSSKSKYPLVVRIYMRMKHVRAWIPRPMRNQNYLFKANPNYAGNSIKPTGTSRNAITTL |
| MY821-3-SAR-97 (GenBank: DQ341367) | B3 | 1997 | Malaysia | SPSAEACGYSDRVAQLTIGNSTITTQEAANIIVGYGEWPSYCSDDDATAVDKPTRPDVSVNRFYTLDTKLWEKSSKGWYWKFPDVLTETGVFGQNAQFHYLYRSGFCIHVQCNASKFHQGALLVAVLPEYVIGTVAGGTGTEDSHPPYKQTQPGADGFELQHPYVLDAGIPISQLTVCPHQWINLRTNNCATIIVPYMNTLPFDSALNHCNFGLLVVPISPLDFDQGATPVIPITITLAPMCSEFAGLRQAVTQGFPTEPKPGTNQFLTTDDGVSAPILPNFHPTPCIHIPGEVRNLLELCQVETILEVNNVPTNATSLMERLRFPVSAQAGKGELCAVFRADPGRDGPWQSTMLGQLCGYYTQWSGSLEVTFMFTGSFMATGKMLIAYTPPGGPLPKDRATAMLGTHVIWDFGLQSSVTLVIPWISNTHYRAHARDGVFDYYTTGLVSIWYQTNYVVPIGAPNTAYIIALAAAQKNFTMKLCKDTSHILQTASIQGDRVADVIESSIGDSVSRALTQALPAPTGQNTQVSSHRLDTGEVPALQAAEIGASSNTSDESMIETRCVLNSHSTAETTLDSFFSRAGLVGEIDLPLEGTTNPNGYANWDIDITGYAQMRRKVELFTYMRFDAEFTFVACTPTGGVVPQLLQYMFVPPGAPKPESRESLAWQTATNPSVFVKLTDPPAQVSVPFMSPASAYQWFYDGYPTFGEHKQEKDLEYGACPNNMMGTFSVRNVGSSKSKYPLVVRIYMRMKHVRAWIPRPMRNQNYLFKANPNYAGNSIKPTGTSRTAITTL |
| TW-2278-2000 (GenBank: MT747901) | B4 | 2000 | Taiwan | SPSAEACGYSDRVAQLTIGNSTITTQEAANIIVGYGEWPSYCSDDDATAVDKPTRPDVSVNRFYTLDTKLWEKSSKGWYWKFPDVLTETGVFGQNAQFHYLYRSGFCIHVQCNASKFHQGALLVAILPEYVIGTVAGGTGTEDSHPPYKQTQPGADGFELQHPYVLDAGIPISQLTVCPHQWINLRTNNCATIIVPYMNTLPFDSALNHCNFGLLVVPISPLDFDQGATPVIPITITLAPMCSEFAGLRQAVTQGFPTEPKPGTNQFLTTDDGVSAPILPNFHPTPCIHIPGEVRNLLELCQVETILEVNNVPTNATSLMERLRFPVSAQAGKGELCAVFRADPGRDGPWQSTMLGQLCGYYTQWSGSLEVTFMFTGSFMATGKMLIAYTPPGGPLPKDRATAMLGTHVIWDFGLQSSVTLVIPWISNTHYRAHARDGVFDYYTTGLVSIWYQTNYVVPIGAPNTAYIIALAAAQKNFTMKLCKDTSHILQTASIQGDRVADVIESSIGDSVSRALTQALPAPTGQNTQVSSHRLDTGEVPALQAAEIGASSNTSDESMIETRCVLNSHSTAETTLDSFFSRAGLVGEIDLPLEGTTNPNGYANWDIDITGYAQMRRKVELFTYMRFDAEFTFVACTPTGGVVPQLLQYMFVPPGAPKPESRESLAWQTATNPSVFVKLTDPPAQVSVPFMSPASAYQWFYDGYPTFGEHKQEKDLEYGACPNNMMGTFSVRTVGSSKSKYPLVVRIYMRMKHVRAWIPRPMRNQNYLFKANPNYAGNSIKPTGTSRTAITTL |
| TW-2792-2002 (GenBank: MT747902) | B4 | 2002 | Taiwan | SPSAEACGYSDRVAQLTIGNSTITTQEAANIIVGYGEWPSYCSDDDATAVDKPTRPDVSVNRFYTLDTKLWEKSSKGWYWKFPDVLTETGVFGQNAQFHYLYRSGFCIHVQCNASKFHQGALLVAILPEYVIGTVAGGTGTEDSHPPYKQTQPGADGFELQHPYVLDAGIPISQLTVCPHQWINLRTNNCATIIVPYMNTLPFDSALNHCNFGLLVVPISPLDFDQGATPVIPITITLAPMCSEFAGLRQAVTQGFPTEPKPGTNQFLTTDDGVSAPILPNFHPTPCIHIPGEVRNLLELCQVETILEVNNVPTNATSLMERLRFPVSAQAGKGELCAVFRADPGRDGPWQSTMLGQLCGYYTQWSGSLEVTFMFTGSFMATGKMLIAYTPPGGPLPKDRATAMLGTHVIWDFGLQSSVTLVIPWISNTHYRAHARDGVFDYYTTGLVSIWYQTNYVVPIGAPNTAYIIALAAAQKNFTMKLCKDTSHILQTASIQGDRVADVIESSIGDSVSRALTQALPAPTGQNTQVSSHRLDTGEVPALQAAEIGASSNTSDESMIETRCVLNSHSTAETTLDSFFSRAGLVGEIDLPLEGTTNPNGYANWDIDITGYAQMRRKVELFTYMRFDAEFTFVACTPTGEVVPQLLQYMFVPPGAPKPESRESLAWQTATNPSVFVKLTDPPAQVSVPFMSPASAYQWFYDGYPTFGEHKQEKDLEYGACPNNMMGTFSVRTVGSSKSKYPLVVRIYMRMKHVRAWIPRPMRNQNYLFKANPNYAGNSIKPTGTSRTAITTL |
| TW-96015-2012 (GenBank: KX267854) | B5 | 2012 | Taiwan | SPSAEACGYSDRVAQLTIGNSTITTQEAANIIVGYGEWPSYCSDDDATAVDKPTRPDVSVNRFYTLDTKLWEKSSKGWYWKFPDVLTETGVFGQNAQFHYLYRSGFCIHVQCNASKFHQGALLVAILPEYVIGTVAGGTGTEDSHPPYKQTQPGADGFELQHPYVLDAGIPISQLTICPHQWINLRTNNCATIIVPYMNTLPFDSALNHCNFGLLVVPISPLDFDQGATPVIPITITLAPMCSEFAGLRQAVTQGFPTEPKPGTNQFLTTDDGVSAPILPNFHPTPCIHIPGEVRNLLELCQVETILEVNNVPTNATSLMERLRFPVSAQAGKGELCAVFRADPGRDGPWQSTMLGQLCGYYTQWSGSLEVTFMFTGSFMATGKMLIAYTPPGGPLPKDRATAMLGTHVIWDFGLQSSVTLVIPWISNTHYRAHARDGVFDYYTTGLVSFWYQTNYVVPIGAPNTAYIIALAAAQKNFTMKLCKDTSHILQTASIQGDRVADVIESSIGDSVSRALTQALPAPTGQNTQVSSHRLDTGEVPALQAAEIGASSNTSDESMIETRCVLNSHSTAETTLDSFFSRAGLVGEIDLPLEGTTNPNGYANWDIDITGYAQMRRKVELFTYMRFDAEFTFVACTPTGQVVPQLLQYMFVPPGAPKPDSRESLAWQTATNPSVFVKLTDPPAQVSVPFMSPASAYQWFYDGYPTFGEHKQEKDLEYGACPNNMMGTFSVRTVGSSKSKYPLVVRIYMRMKHVRAWIPRPMRNQNYLFKANPNYAGNSIKPTGTSRTAITTL |
| TW-6001-2018 (GenBank: MT747903) | B5 | 2018 | Taiwan | SPSAEACGYSDRVAQLTIGNSTITTQEAANIIVGYGEWPSYCSDDDATAVDKPTRPDVSVNRFYTLDTKLWEKSSKGWYWKFPDVLTETGVFGQNAQFHYLYRSGFCIHVQCNASKFHQGALLVAILPEYVIGTVAGGTGTEDSHPPYKQTQPGADGFELQHPYVLDAGIPISQLTICPHQWINLRTNNCATIIVPYMNTLPFDSALNHCNFGLLVVPISPLDFDQGATPVIPITITLAPMCSEFAGLRQAVTQGFPTEPKPGTNQFLTTDDGVSAPILPNFHPTPCIHIPGEVRNLLELCQVETILEVNNVPTNATSLMERLRFPVSAQAGKGELCAVFRADPGRDGPWQSTMLGQLCGYYTQWSGSLEVTFMFTGSFMATGKMLIAYTPPGGPLPKDRATAMLGTHVIWDFGLQSSVTLVIPWISNTHYRAHARDGVFDYYTTGLVSIWYQTNYVVPIGAPNTAYIIALAAAQKNFTMKLCKDTSHILQTASIQGDRVADVIESSIGDSVSRALTRALPAPTGQNTQVSSHRLDTGEVPALQAAEIGASSNTSDESMIETRCVLNSHSTAETTLDSFFSRAGLVGEIDLPLEGTTNPNGYANWDIDITGYAQMRRKVELFTYMRFDAEFTFVACTPTGEVVPQLLQYMFVPPGAPKPDSRESLAWQTATNPSVFVKLTDPPAQVSVPFMSPASAYQWFYDGYPTFGEHKQEKDLEYGACPNNMMGTFSVRTVGSSKSKYPLVVRIYMRMKHVRAWIPRPMRNQNYLFKANPNYAGNSIKPTGASRTAITTL |
| TW-901-2019 (GenBank: MT747904) | B5 | 2019 | Taiwan | SPSAEACGYSDRVAQLTIGNSTITTQEAANIIVGYGEWPSYCSDDDATAVDKPTRPDVSVNRFYTLDTKLWEKSSKGWYWKFPDVLTETGVFGQNAQFHYLYRSGFCIHVQCNASKFHQGALLVAILPEYVIGTVAGGSGTEDSHPPYKQTQPGADGFELQHPYVLDAGIPISQLTICPHQWINLRTNNCATIIVPYMNTLPFDSALNHCNFGLLVVPISPLDFDQGATPVIPITITLAPMCSEFAGLRQAVTQGFPTEPKPGTNQFLTTDDGVSAPILPNFHPTPCIHIPGEVRNLLELCQVETILEVNNVPTNATSLMERLRFPVSAQAGKGELCAVFRADPGRDGPWQSTMLGQLCGYYTQWSGSLEVTFMFTGSFMATGKMLIAYTPPGGPLPKDRATAMLGTHVIWDFGLQSSVTLVIPWISNTHYRAHARDGVFDYYTTGLVSIWYQTNYVVPIGAPNTAYIIALAAAQKNFTMKLCKDTSHILQTASIQGDRVADVIESSIGDSVSRALTRALPAPTGQNTQVSSHRLDTGEVPALQAAEIGASSNTSDESMIETRCVLNSHSTAETTLDSFFSRAGLVGEIDLPLEGTTNPNGYANWDIDITGYAQMRRKVELFTYMRFDAEFTFVACTPTGQVVPQLLQYMFVPPGAPKPDSRESLAWQTATNPSVFVKLTDPPAQVSVPFMSPASAYQWFYDGYPTFGEHKQEKDLEYGACPNNMMGTFSVRTVGSSKSKYPLVVRIYMRMKHVRAWIPRPMRNQNYLFKANPNYAGNSIKPTGASRTVITTL |
| Tainan/5079/98 (GenBank: AF177911) | Cox A16 | 1998 | Taiwan | SPSAEACGYSDRVAQLTIGNSTITTQEAANIVIAYGEWPEYCPDTDATAVDKPTRPDVSVNRFFTLDTKSWAKDSKGWYWKFPDVLTEVGVFGQNAQFHYLYRSGFCVHVQCNASKFHQGALLVAVLPEYVLGTIAGGTGNENSHPPYATTQPGQVGAVLTHPYVLDAGIPLSQLTVCPHQWINLRTNNCATIIVPYMNTVPFDSALNHCNFGLLVVPVVPLDFNAGATSEIPITVTIAPMCAEFAGLRQAVKQGIPTELKPGTNQFLTTDDGVSAPILPGFHPTPPIHIPGEVHNLLEICRVETILEVNNLKTNETTPMQRLCFPVSVQSKTGELCAAFRADPGRDGPWQSTILGQLCRYYTQWSGSLEVTFMFAGSFMATGKMLIAYTPPGGNVPADRITAMLGTHVIWDFGLQSSVTLVVPWISNTHYRAHARAGYFDYYTTGIITIWYQTNYVVPIGAPTTAYIVALAAAQDNFTMKLCKDTEDIEQTANIQGDPIADMIDQTVNNQVNRSLTALQVLPTAADTEASSHRLGTGVVPALQAAETGASSNASDKNLIETRCVLNHHSTQETAIGNFFSRAGLVSIITMPTTGTQNTDGYVNWDIDLMGYAQLRRKCELFTYMRFDAEFTFVVAKPNGELVPQLLQYMYVPPGAPKPTSRDSFAWQTATNPSVFVKMTDPPAQVSVPFMSPASAYQWFYDGYPTFGEHLQANDLDYGQCPNNMMGTFSIRTVGTEKSPHSITLRVYMRIKHVRAWIPRPLRNQPYLFKTNPNYKGNDIKCTSTSRDKITTL |
