# Supplementary material for: Emergence of genotype C1 Enterovirus A71 and its link with antigenic variation of virus in Taiwan
Source: PLoS Pathog. 2020 Sep 16;16(9):e1008857. doi: 10.1371/journal.ppat.1008857 (PMC7521691; doi:10.1371/journal.ppat.1008857)
Supplement: S3 Table — (PDF) [file ppat.1008857.s006.pdf]

S3 Table. Convalescent sera from patients with acute EV-A71 infections, 2014-2017.

Serum	Age (years)	Severe neurological involvement[a]	IVIG usage	Exposed EV-A71 (genotype)
EV-A71-1	1.4	-	-	B5
EV-A71-2	3.9	-	-	B5
EV-A71-3	0.7	yes	yes	C4
EV-A71-4	4.8	-	-	B5
EV-A71-5	3.8	-	-	B5
EV-A71-6	3.7	yes	yes	C4
EV-A71-7	3.4	-	-	B5
EV-A71-8	3.8	-	-	B5
EV-A71-9	3.2	-	-	B5
EV-A71-10	5.0	-	-	C4
EV-A71-11	5.0	-	-	B5
EV-A71-12	4.4	-	-	B5
EV-A71-13	0.8	-	-	B5
EV-A71-14	5.8	-	-	B5
EV-A71-15	2.0	-	-	B5
EV-A71-16	1.9	-	-	B5
EV-A71-17	3.1	-	-	B5
EV-A71-18	4.0	-	-	B5
EV-A71-19	2.7	-	-	B5
EV-A71-20	1.4	-	-	B5
EV-A71-21	5.1	-	-	B5
EV-A71-22	2.3	-	-	B5
EV-A71-23	1.5	-	-	B5

[a] Severe neurological involvement was defined as the presence of encephalitis (such as changes in consciousness, seizure, ataxia, or cranial nerve palsy) and/or autonomic nervous system dysregulation (such as tachycardia, hyperthermia, profuse sweating, or transient hypertension) [1].

Abbreviations; IVIG, intravenous immunoglobulin.
