# Supplementary material for: Emergence of genotype C1 Enterovirus A71 and its link with antigenic variation of virus in Taiwan
Source: PLoS Pathog. 2020 Sep 16;16(9):e1008857. doi: 10.1371/journal.ppat.1008857 (PMC7521691; doi:10.1371/journal.ppat.1008857)
Supplement: S4 Table — (PDF) [file ppat.1008857.s007.pdf]

S4 Table. Primers for sequencing of VP1, VP2 and VP3 of EV-A71 in the study.

Genotype	Forward (5'-3')	Reverse (5'-3')
C1		
VP1	GCAGCAGCCCAGAAAAA	AAGGTTTGCCCAATCATTGTG
VP2	ACAGAGCCTTAAACAAGATCCAGAT	AAAGTTCGGCAGGATGGGTGC
VP3	TTCGACCAAGGAGCGACAC	CTGTAGGCGCTGGTAAAGC
C4		
VP1	GCGGCAGCCCAAAAGAA	AAGATTTGCCCAATCATTGTG
VP2	ACAGAGTCTCAAGCAGGAT	GAAGTTTGGTAGAATAGGTGC
VP3	TACGACCAAGGAGCGACGC	CTGTGGGTGCTGGTAGAGC
B5		
VP1	ACCATGAAACTCTGCAAGG	GAAAAACTGACTGGGTAGTG
VP2	ACAGAGCCTCAAACAAGAC	GAAATTTGGCAGAATGGGTGC
VP3	ATCACTCTAGCTCCAATGTGCTC	CTACTCACACTGTCTCCTATAGAGC
